# Multitask ATPases (NBDs) of bacterial ABC importers type I and their interspecies exchangeability

**DOI:** 10.1038/s41598-020-76444-0

**Published:** 2020-11-11

**Authors:** Francisco Leisico, Lia M. Godinho, Inês C. Gonçalves, Sara P. Silva, Bruno Carneiro, Maria J. Romão, Teresa Santos-Silva, Isabel de Sá-Nogueira

**Affiliations:** 1 Microbial Genetics Laboratory, UCIBIO, Departamento de Ciências da Vida, Faculdade de Ciências E Tecnologia, Universidade NOVA de Lisboa, Quinta da Torre, 2829-516 Caparica, Portugal; 2 XTAL – Macromolecular Crystallography Laboratory, UCIBIO, Departamento de Química, Faculdade de Ciências E Tecnologia, Universidade NOVA de Lisboa, Quinta da Torre, 2829-516 Caparica, Portugal

**Keywords:** Microbiology, Molecular biology, Structural biology

## Abstract

ATP-binding cassette (ABC) type I importers are widespread in bacteria and play a crucial role in its survival and pathogenesis. They share the same modular architecture comprising two intracellular nucleotide-binding domains (NBDs), two transmembrane domains (TMDs) and a substrate-binding protein. The NBDs bind and hydrolyze ATP, thereby generating conformational changes that are coupled to the TMDs and lead to substrate translocation. A group of multitask NBDs that are able to serve as the cellular motor for multiple sugar importers was recently discovered. To understand why some ABC importers share energy-coupling components, we used the MsmX ATPase from *Bacillus subtilis* as a model for biological and structural studies. Here we report the first examples of functional hybrid interspecies ABC type I importers in which the NBDs could be exchanged. Furthermore, the first crystal structure of an assigned multitask NBD provides a framework to understand the molecular basis of the broader specificity of interaction with the TMDs.

## Introduction

ATP-binding cassette (ABC) transporters are found in all three domains of life, from bacteria to humans, and constitute a large superfamily of membrane translocator proteins of a wide variety of molecules, such as ions, amino acids, sugars, lipids, peptides, proteins and antibiotics^1,2^. ABC exporters and importers share a core structural design composed of at least two intracellular nucleotide-binding domains (NBDs) and two transmembrane domains (TMDs) embedded in the lipid bilayer that form the translocation pore, and the same mechanism of ATP binding and catalysis to energize the movement of substrates across the membrane^3^. In the assembled transporter, NBDs are present as a dimer, with the ATP binding site of each monomer facing the other. The amino acid residues involved in ATP binding and hydrolysis are highly preserved and are positioned in recognized motifs that characterize the superfamily of ABC proteins: Walker A and B and canonical motifs, and the Q, H, and D loops^4^. While exporters are present in all domains of life, importers exist only in bacteria and plants^5–7^. In bacteria, type I and II importers rely on a substrate-binding protein (SBP), which captures the substrate and transfers it to the TMDs^8,9^; type I permeases import mostly metabolites such as sugars and amino acids, whereas type II substrates are trace non-metabolites such as metal chelates and vitamins. The two groups of transporters share the same NBD fold but their TMDs are totally different: the two TMDs of type I importers are either identical or structurally similar (like the prototype maltose transporter MalEFGK_2_ from *Escherichia coli*^10^), with a core membrane topology of five TM helices per TMD, while those of type II have two identical TMDs each containing ten TM helices. In addition, these two different types of importers display mechanistic differences^2,4,7,8,11^.

In bacterial genomes, the components of ABC transporters are generally encoded by genes in operons or clustered together and are often subjected to a coordinated regulation of expression. In the chromosome of the Gram-positive model organism *B. subtilis,* at least seven incomplete ABC carbohydrate uptake systems lacking a gene encoding the energy-coupling component (NBD) were identified^12^. An orphan ATPase, MsmX, located in a distinct locus, was proposed as their missing NBD^12^. MsmX was shown to energize six of these transporters, being involved in the uptake of maltodextrins (MdxEFG^13^), arabino-oligossacharides (AraNPQ^14^), galacto-oligossacharides (GanSPQ^15^), galacturonic acid oligomers and/or rhamnose-galacturonic acid disaccharides (YesOPQ and YtcPQ-YteP^15^ and melibiose (MelECD^16^). Due to its broad capacity to interact and energize multiple TMDs of distinct transport systems, MsmX was named multitask ATPase^14^. Similarly, the genome of the pathogen *Streptococcus pneumoniae* TIGR4 encodes six incomplete carbohydrate ABC importers, lacking a gene encoding the ATPase required to energize the transport, and it was shown that the orphan ATPase MsmK, encoded by a gene at a distinct site in the genome, energizes four of the six importers^17,18^. Although other multitask ABC ATPases have been identified in other bacteria such as *Streptomyces lividans*^19^ and *Streptococcus suis*^20^, the ability to interact with and energize multiple transporters is rather uncommon among ABC NBDs. In fact, to date, proteins with this ability have been exclusively reported in bacterial ABC sugar importers of the subfamily of carbohydrate uptake transporter 1 (CUT1), that transport a variety of di‐, tri‐ and higher oligosaccharides, as well as polyols^21,22^.

Here we address the question of why some ABC importers share energy generating components. First, we investigate how well conserved this phenomenon is among bacteria by studying intra- and interspecies functional exchangeability of NBDs among species of Gram-negative and Gram-positive bacteria. Secondly, we expand the identification of other putative multitask ATPases outside the Firmicutes phylum, using a potential signature sequence motif derived from bioinformatic analysis and site-directed mutagenesis. Finally, we solve the crystal structure of the *B. subtilis* multitask ATPase MsmX K43A; the crystals diffracted up to 1.67 Å resolution and based on comparative analysis we hypothesize about sequence and structural features involved in the broader specificity of NBDs.

## Results

### Interspecies functional exchangeability of multitask ATPases (NBDs) from type I ABC transport systems

**Figure 1:**
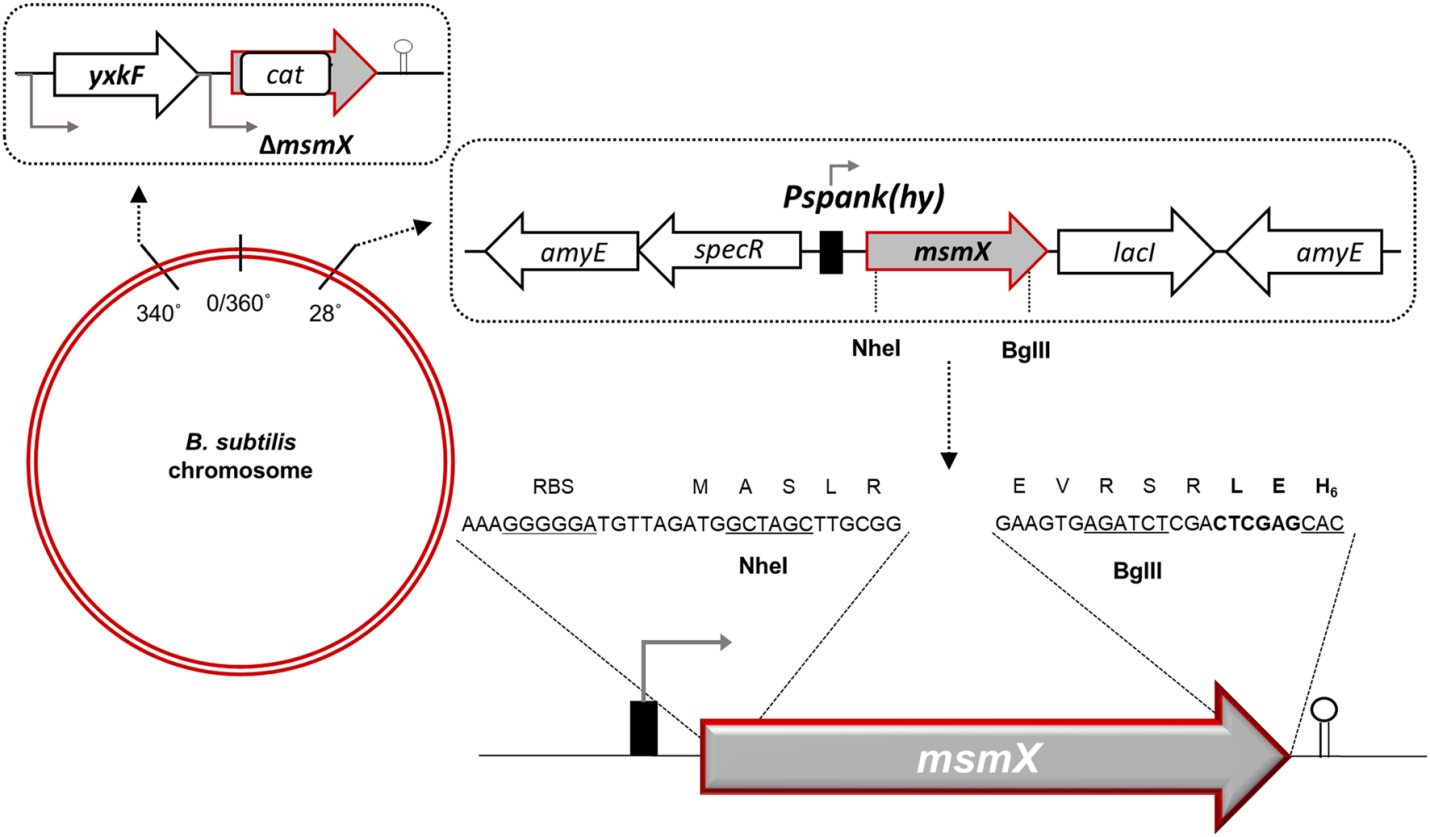
Genetic system for conditional expression of NBDs in *B. subtilis*. A copy of the *msmX* gene under the control of the regulable promoter Pspank(hy) was integrated, by a double-recombinational event, at the *amyE* locus of the *B. subtilis* chromosome (top right) in a *msmX-null* mutant genetic background (top left). The sequence of the *msmX* allele, at the 5′- and 3′-end, was modified to accommodate several features (bottom right). In the C-terminal region of MsmX a LEH_6_-tag was added to enable detection of protein production by western blot. Moreover, two unique restriction sites were introduced in the coding region, *Nhe*I (5′-end) and *Bgl*II (3′-end), to facilitate sub-cloning of the different NBDs. The functionality of distinct multitask ATPases alleles as well as mutagenized *msmX* is determined by their ability to complement the role of MsmX as energy generator of distinct sugar importers.

To characterize the energy-coupling component of bacterial ABC transporters, and to evaluate their intra- and interspecies exchangeability, we constructed a genetic system in *B. subtilis* for regulated expression of the *msmX* allele or its homologs in *trans* (Fig. 1). The functionality of distinct NBDs alleles was determined by their capacity to substitute MsmX, as energizer of different carbohydrates importers, in a *B. subtilis msmX*-null mutant. Furthermore, the NBDs were expressed and produced with a His_6_-tag placed at the C-terminus, which allowed the determination of the protein accumulation *in B. subtilis* cells.

**Figure 2:**
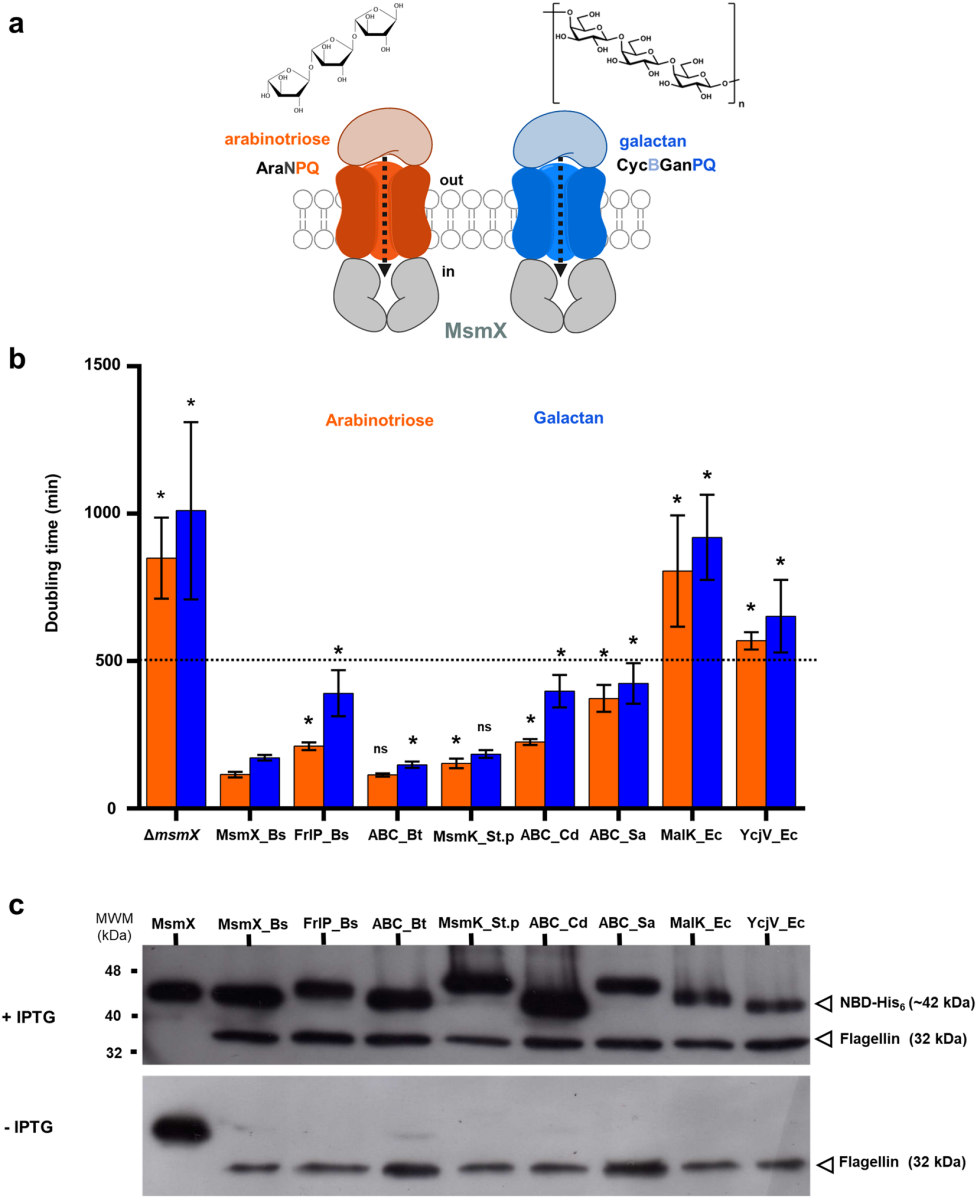
Functionality of MsmX homologs tested by their ability to support growth in distinct oligosaccharides. (**a**) Schematic representation of the AraNPQ and GanSPQ(CycBGanPQ) transport systems of *B. subtilis*, which import arabino- oligomers (orange) and galacto-oligomers (blue), respectively. (**b**) Growth kinetics parameters of the *msmX*-null mutant *B. subtilis* strain (IQB495) and the strains carrying different NBD alleles, ISN10(*msmX_Bs*), ISN15(*frlP_Bs*), ISN11(*ABC_Bt*), ISN12(*msmK_St.p*), ISN25(*ABC_Cd*), ISN13(*ABC_Sa*), ISN14(*malK_Ec*), ISN17(*ycjV_Ec*), were determined in the presence of arabinotriose (orange) or galactan (blue). The doubling time of each strain in the presence of the inducer IPTG is shown. A value above 500 min (dashed line) is considered no growth (see Supplementary Table 1). At least three independent experiments were performed in each condition and error bars indicate the standard deviation of the mean. Statistical significance between doubling time of each strain bearing a MsmX homolog and the strain ISN10 (*msmX_Bs*) is indicated (*p < 0.05; ns: not significant) and was obtained using the R software version 3.6.2 (https://www.r-project.org/). (**c**) Western blot analysis of NBD accumulation in total cell extracts of each strain grown in the presence (top panel) or absence (bottom panel) of IPTG. Purified MsmX-His_6_ (0.25 μg) was loaded on the first lane. Low Molecular Weight, Protein Marker II (NZYTech) was used and is partially represented (MWM). NBDs and flagellin (loading control) detection are indicated by open arrowheads. Uncropped images of these blots are presented in Supplementary Fig. 5.

Based on the sequence identity (shown in parenthesis), we selected known and putative NBDs from Gram-positive and Gram-negative bacteria: *B. subtilis* FrlP(YurJ (58%)); *B. thuringiensis* ABC_Bt (74%); *Streptococcus pneumoniae* MsmK (64%); *Staphylococcus aureus* ABC_Sa (66%); *Clostridioides difficile* ABC_Cd (57%); and *Escherichia coli* YcjV (64%) and MalK (45%). The analysis of the respective genomes revealed that the putative NBDs are either orphan or clustered together with other components of an ABC importer (Supplementary Fig. 1). Among the targeted NBDs, only MsmK from *Strep. pneumoniae*^17,18^ and MalK^23^ from *E. coli* have been characterized so far. MalK was selected because MalEFGK_2_ is the prototype of ABC type I sugar importers and its 3D structure has been determined before^10^. The functionality of these distinct ATPases alleles was determined by their ability to substitute MsmX as the NBD of two different sugar importers—AraNPQ^14,15^ and GanSPQ^15,24^ in a *B. subtilis msmX*-null mutant (Fig. 2a). By determining the growth kinetic parameters in the presence of the substrates of each importer as sole carbon and energy source, arabinose-oligomers and galactose-oligomers, respectively, we were able to assess the degree of efficiency of each NBD to substitute MsmX. The results are summarized in Fig. 2b (detailed data in Supplementary Table 1), where a higher doubling time reflects lower functional ability of the tested NBD to energize the respective importer AraNPQ (arabinotriose) or GanSPQ (galactan). This indicates that all NBDs tested were able to complement *msmX*-null mutation, except YcjV and MalK from *E. coli*. The ABC_Bt from *B. thuringiensis* is the more efficient NBD in its ability to substitute MsmX and energize both AraNPQ and GanSPQ, with a doubling time of 113.8 ± 6.2 min (arabinotriose) and 148.6 ± 10.9 min (galactan) when compared with the strain bearing the *msmX* allele, which has a doubling time of 115.4 ± 9.3 min and 172.4 ± 9.3 min, respectively (Fig. 2b and Supplementary Table 1). On the other hand, the ABC_Sa from *Staph. aureus* is the less efficient 373.7 ± 45.8 min (arabinotriose) and 424.1 ± 68.6 min (galactan). YcjV and MalK from *E. coli* do not support growth in arabinotriose or galactan on a *msmX*-null mutant genetic background.

In addition, the accumulation of the NBDs expressed in *B. subtilis* was determined by western blot analysis, using a His_6_-tag antibody (Fig. 2c). Although YcjV and MalK are not able to function as NBDs of the importers AraNPQ and GanSPQ, they both accumulate in the *B. subtilis* cells, which suggests that protein misfolding/aggregation or low expression levels can be excluded (Fig. 2c). Furthermore, biochemical characterization shows that YcjV and MalK retain their ATPase activity (Supplementary Fig. 2). The results indicate functional exchangeability of multitask ATPases among Gram-positive bacteria within the Firmicutes phylum.

### A putative signature sequence motif for bacterial multitask ATPases

**Figure 3:**
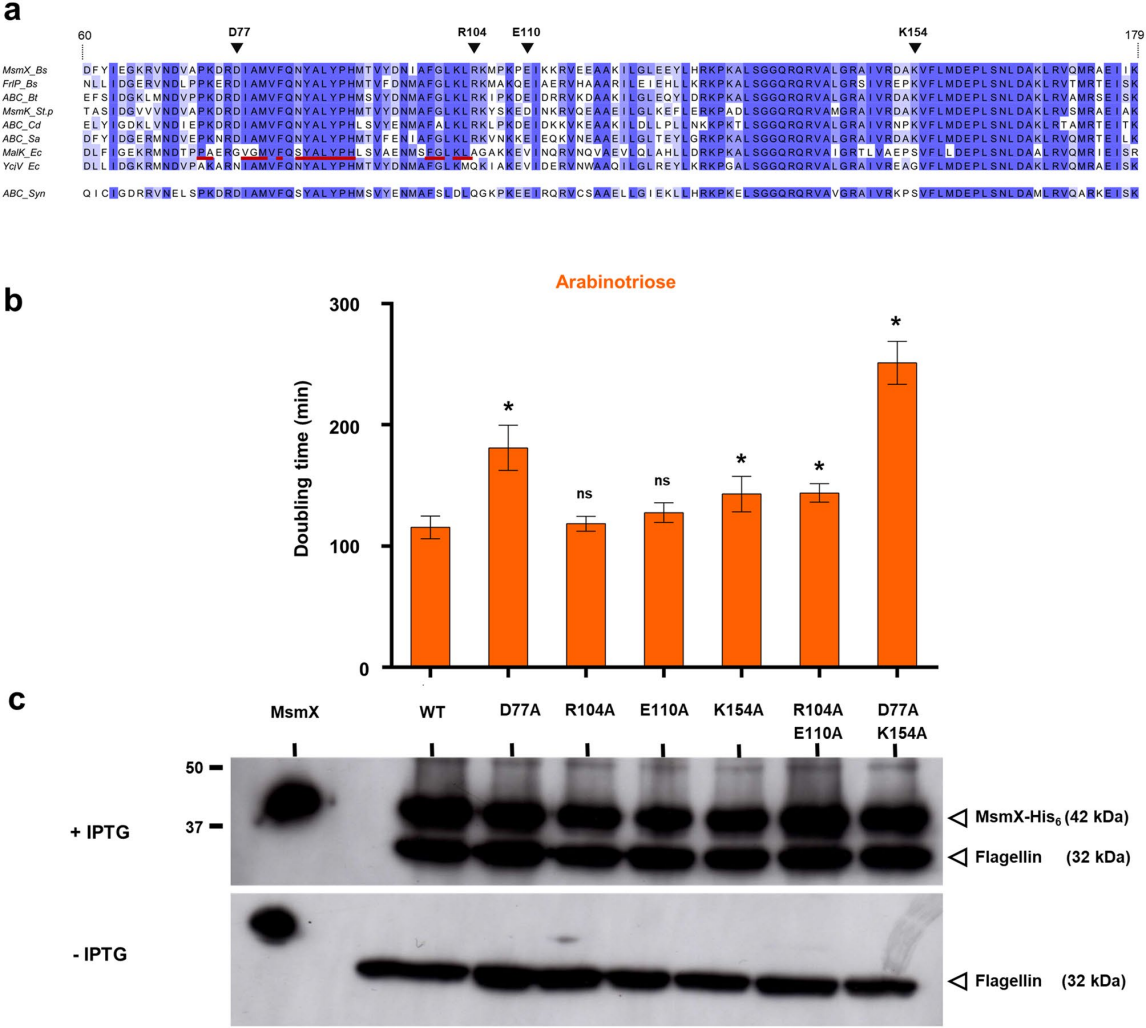
Analysis of TMD-NBD contacting region and site-directed mutagenesis of MsmX. (**a**) Primary sequence alignment of the TMD-NBD contacting region of MsmX homologs. Conserved residues are depicted in dark blue and amino acids selected for mutagenesis are indicated by solid black arrowheads. MalK residues involved in interactions with the TMDs of the *E. coli* maltose transporter MalFGK_2_ are underlined in red. Primary sequence alignment was obtained using Jalview Version: 2.11.1.0 (https://www.jalview.org). (**b**) Functionality of the different *msmX*-mutant alleles was assessed by determination of growth kinetic parameters in minimal medium with arabinotriose as the sole carbon and energy source. The doubling time of each strain in the presence of the inducer IPTG is shown (see also Supplementary Table 2). At least three independent experiments were performed for each strain and error bars indicate the standard deviation of the mean. Statistical significance of the doubling time of each strain bearing a MsmX mutant variant, with single D77A, R104A, E110A, K154A, or double R104A/E110A, D77A/K154A mutations, compared to the doubling time of strain ISN10 carrying the wild-type (WT) allele is indicated (*p < 0.05; ns: not significant) and was obtained using the R software version 3.6.2 (https://www.r-project.org/). **c** Western blot analysis of MsmX and MsmX mutant variants accumulation in total cell extracts of each strain grown in the presence (top panel) or absence (bottom panel) of IPTG. Purified MsmX-His_6_ (0.25 μg) was loaded on the first lane. Precision Plus Protein All Blue Prestained Protein Standard (BioRad) was used and is partially represented (MWM). MsmX mutants and flagellin (loading control) detection are indicated by open arrowheads. Uncropped images of these blots are presented in Supplementary Fig. 6.

A multiple sequence alignment of the NBDs examined in this study highlighted amino acids conserved in all sequences from different species (*B. subtilis*, *B. thuringiensis*, *Strep. pneumoniae*, *Staph. aureus* and *C. difficile*) capable to achieve the role of MsmX but not present in proteins unable to accomplish its function, as MalK and YcjV from *E. coli* (among the conserved residues, the only exception is E110 that in MsmK from *Strep. pneumoniae* is replaced by an aspartate) (Fig. 3a). The majority of these residues is located in the predicted region for the NBD/TMD interaction, between positions 60 and 179 of MsmX. To test their relevance upon complex formation and substrate import, we targeted D77, R104, E110, K154 in MsmX. These amino acids were exchanged by alanines, using site-directed mutagenesis, and their effect in the fitness of each MsmX mutant was analized in vivo, as described above. The results of the growth kinetic parameters revealed a different degree of aptitude of each NBD variant to act as energy motor of the AraNPQ transporter (Fig. 3b and Supplementary Table 2). Regarding single mutations, the variant D77A displayed the highest negative impact in transport/growth (180.9 ± 18.7 min doubling time) when compared with the wild-type (115.4 ± 9.3 min doubling time). In addition, the double-mutations D77A/K154A and R104A/E110A were also tested, revealing a cumulative effect when compared to the single mutants (Fig. 3b), with a more pronounced negative impact in cell growth in the first pair D77A/K154A (251.2 ± 17.8 min doubling time). The latter was overexpressed, purified, and shown to retain ATPase activity (Supplementary Fig. 2). Since the intracellular accumulation of all MsmX variants is similar, as assessed by western blot (Fig. 3c), data from single and double mutations suggest that all targeted amino acids are important for the NBD-TMD contact and/or proper conformation of the protein complex required to drive the transport of substrate.

### Interspecies functional exchangeability of NBDs beyond the Firmicutes phylum

**Figure 4:**
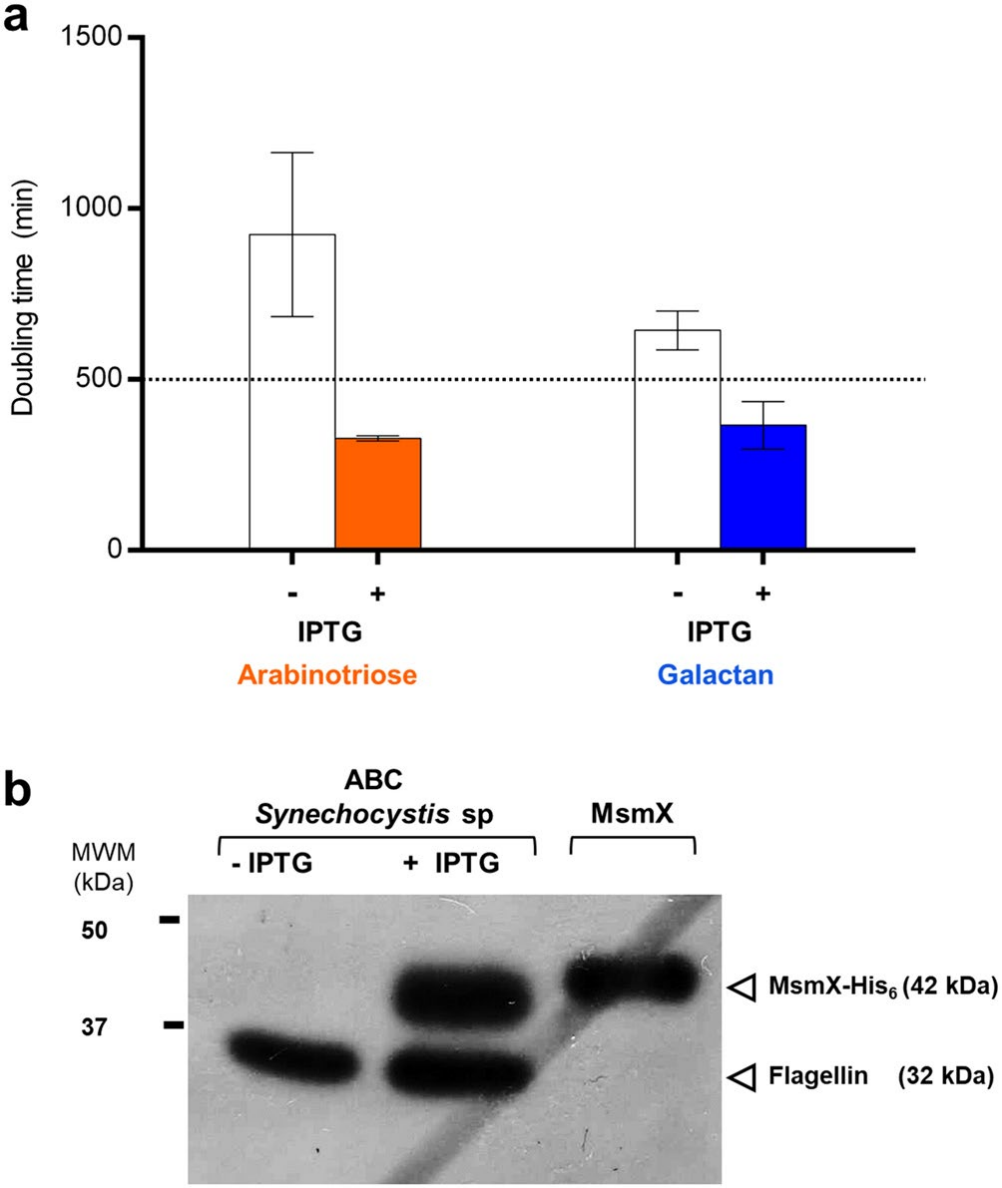
Exchangeability of NBDs outside the Firmicutes phylum. (**a**) Growth kinetics parameters of a *msmX*-null *B. subtilis* strain (ISN58) harboring the ABC_Syn NBD from *Synechocystis sp.* PCC6803 was determined in minimal medium in the presence of the arabinotriose (orange) or galactan (blue) as sole carbon and energy source. The doubling time of the strain in the absence (white bars) or presence (colored bars) of the inducer IPTG is shown. At least three independent experiments were performed for each strain and error bars indicate the standard deviation of the mean. A value above 500 min (dashed line) is considered no growth. **b**) Western blot analysis of NBD accumulation in total cell extracts grown in the absence (-) or presence ( +) of IPTG. Purified MsmX-His_6_ (0.25 μg) was loaded on the last lane. Precision Plus Protein All Blue Prestained Protein Standard (BioRad) was used and is partially represented (MWM). NBDs and flagellin (loading control) detection are indicated by open arrowheads. Uncropped image of this blot is presented in Supplementary Fig. 7.

To check the presence of putative multitask ATPases in other prokaryotic phyla, a bioinformatic search of genome databases was conducted using the same NBD-TMD interaction region of MsmX (residues 60–179). Among the potential hits beyond the Firmicutes phylum, a putative NBD from an ABC transporter from *Synechocystis sp.*, was selected and integrated into the chromosome of the *msmX*-null mutant to test its functionality in *B. subtilis*, as described above. This putative NBD displays sequence identity of 51% with *B. subtilis* MsmX in the total extension of both proteins, while the identity in the targeted region encompassing potential NBD-TMD interactions is 71% (ABC_Syn; Fig. 3a, bottom). Growth experiments and western blot analysis revealed that ABC_Syn accumulates in *B. subtilis* cells and is able to substitute MsmX as the NBD of the two *B. subtilis* sugar importers AraNPQ and GanSPQ (Fig. 4). The doubling time of the strain harboring the hybrid importers is 328.5 ± 7.4 min (arabinotriose) and 366.3 ± 69.3 (galactan) (see Supplementary Table 1), twice the time of the strain bearing the *msmX* allele (Fig. 2b). This result extends the interspecies exchangeability of NBDs to the Cyanobacteria phylum and to Gram-negative bacteria.

### The first crystal structure of a multitask ATPase

The MsmX protein from *B. subtilis* was recombinantly overexpressed for structural characterization. During purification, MsmX wild-type showed high propensity to aggregate and high content of nucleic acids. This high level of contamination and aggregation was decreased when using the MsmX K43A variant. K43 is involved in ATP coordination and catalysis, interacting with the β- and γ-phosphate groups. Its replacement by an alanine resulted in 80% decreased ATPase activity (Supplementary Fig. 3), impairing the normal function of MsmX to energize the AraNPQ transporter in *B. subtilis* (Supplementary Table 2). This mutant crystallized in I222 space group, with 1 molecule in the asymmetric unit. The crystal structure was solved at 1.67 Å resolution and the final electron density map allowed to model a detailed refined structure, which comprises all residues of MsmX K43A, except the N-terminus methionine.

Interestingly, most crystal structures of NBDs from ABC transporters show a dimer in the asymmetric unit, promoted either by the C-terminal domains or the presence of ATP (or analogs). This is not the case for *B. subtilis* MsmX, that crystallized as a monomer with one molecule in the asymmetric unit, and very few contacts with symmetry related molecules, as indicated by analyzing the protein interface using PISA server^25^. In fact, MsmX is eluted as a monomer in the last step of purification by size exclusion chromatography, suggesting that the protein is also in the monomeric form during purification.

**Figure 5:**
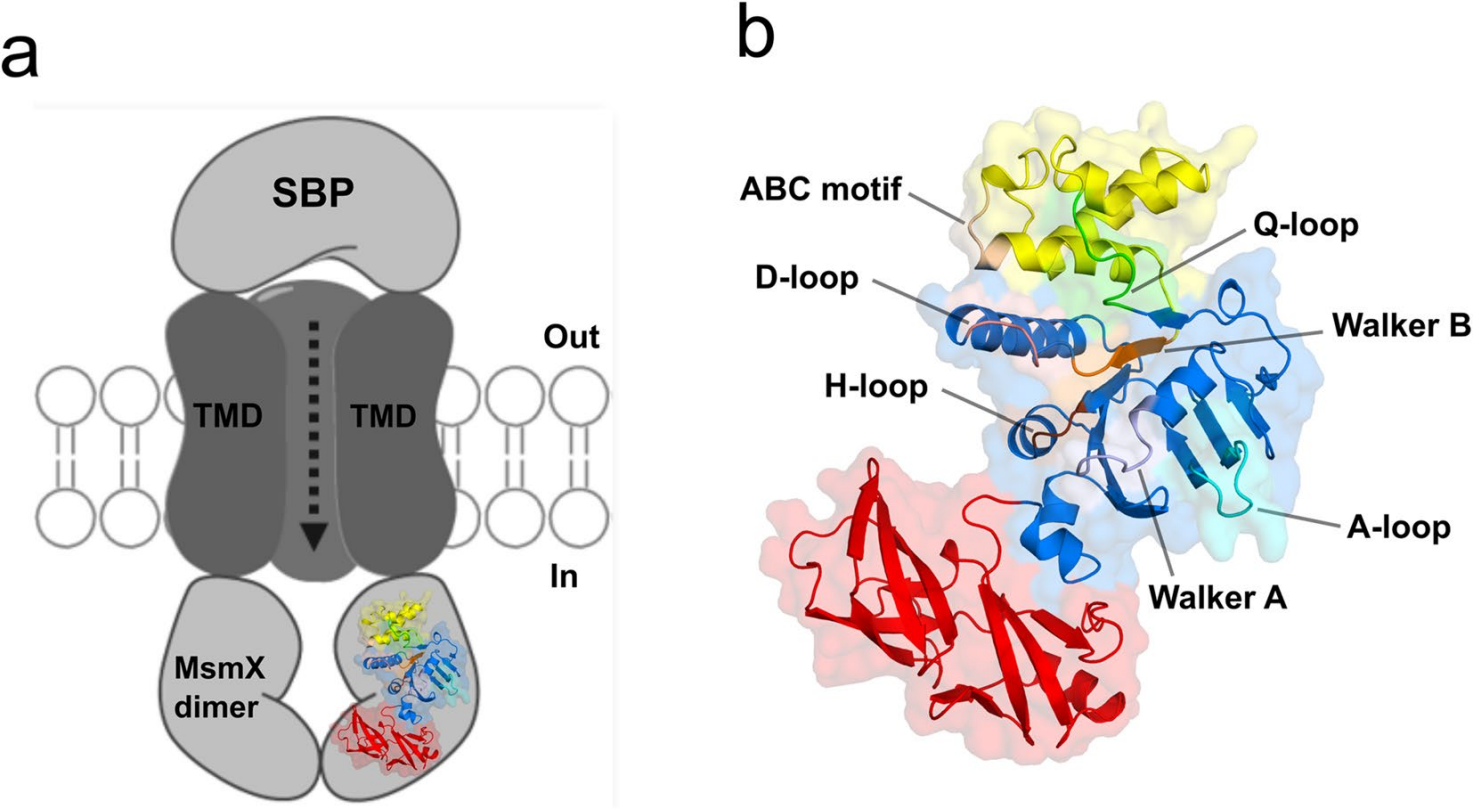
Crystal structure of the multitask MsmX K43A from *B. subtilis*. (**a**) Schematic representation of an ABC importer energized by the MsmX dimer, including the transmembrane domains (TMDs) and the substrate binding protein (SBP). (**b**) Cartoon representation of MsmX K43A crystal structure. Functional domains and motifs characteristic of NBDs from ABC transporters are in different colors: RecA-like domain in blue; α-helical domain in yellow; regulatory C-terminal domain in red; A-loop in cyan; Walker A in grey; Q-loop in green; ABC motif in light brown; Walker B in orange; D-loop in salmon; H-loop in brown. Figure obtained with The PyMOL Molecular Graphics System, Version 2.0.5 Schrodinger, LLC (https://pymol.org/2/).

**Figure 6:**
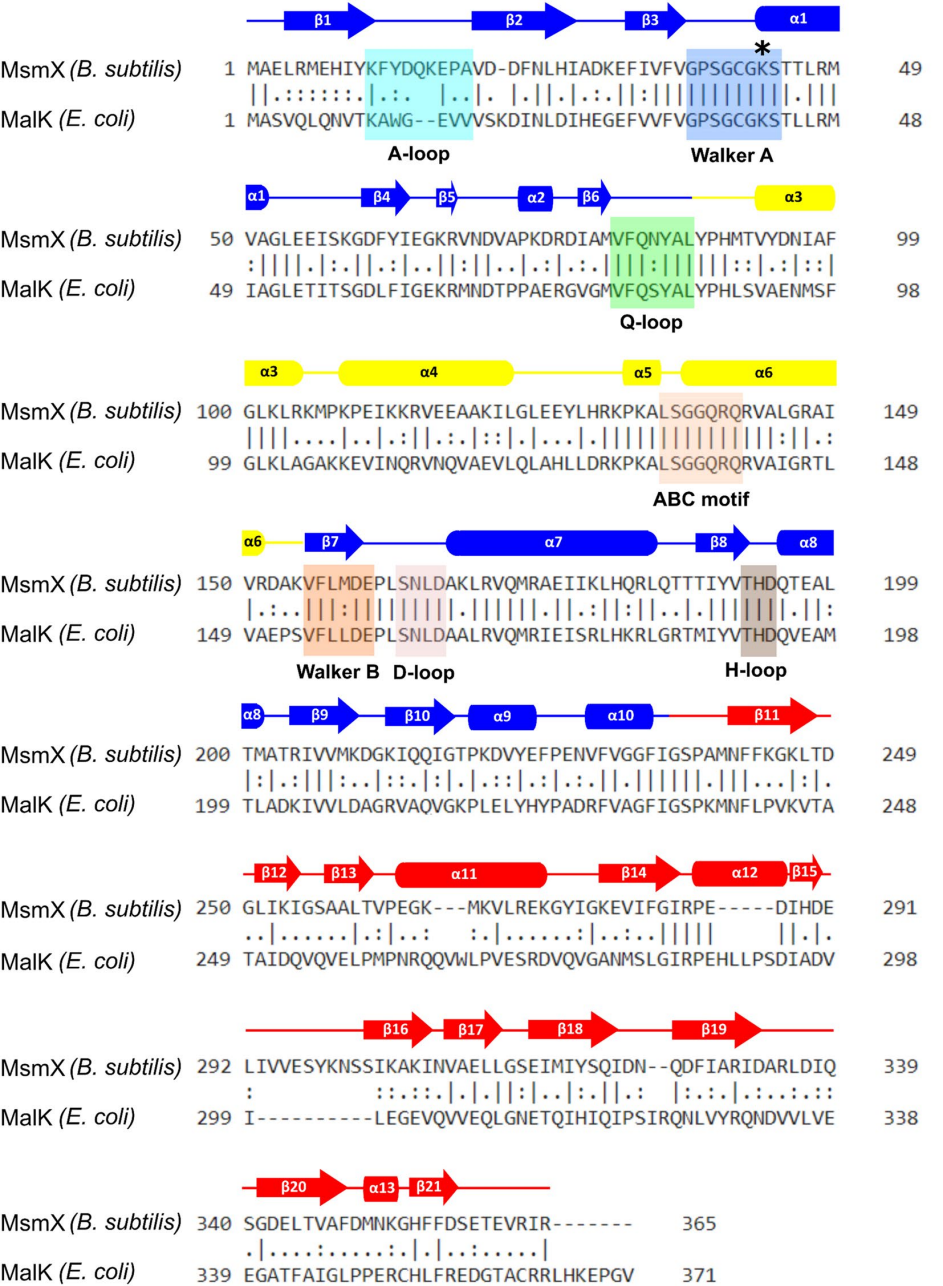
Sequence alignment of *B. subtilis* MsmX and *E. coli* MalK proteins. Primary sequences of MsmX and MalK (Uniprot code P68187) were aligned using EMBOSS Needle (https://www.ebi.ac.uk/Tools/psa/emboss_needle/). Functional motifs characteristics of NBDs are highlighted and colored according to the structure of *B. subtilis* MsmX K43A presented in Fig. 5. Secondary structure elements were assigned using STRIDE software (https://webclu.bio.wzw.tum.de/stride/35) and are represented above the MsmX sequence. K43 mutation is marked with *.

MsmX K43A shares the fold of well-studied NBDs of ABC type I importers (Fig. 5a,b)^8^. As shown in Fig. 5b, MsmX N-terminal domain presents the general α/β-type ATPase domain fold, which comprises the RecA-like domain (residues 2–87 and 155–235, in blue) and the α-helical domain (residues 88–154, in yellow). The regulatory C-terminal domain (residues 236–365, in red) forms a mixed barrel with 3 α-helices and 11 β-strands (Figs. 5b, 6). MsmX K43A has a high structural similarity with the MalK protein from *E. coli* and *Pyrococcus horikoshii*, as derived by PDBeFold^26^(Supplementary Table 3). These proteins have been crystallized in the open, semi-open and closed conformations, corresponding to the ligand-free, ADP + Mg^2^^+^ or ATP bound forms, respectively^23,27–31^. The presence of ATP is mandatory to achieve the functional closed conformation of the MalK dimer; the MalFGK_2_ complex achieves the pre-translocation state in the presence of the Maltose Binding Protein and maltose, while the outward open form when, additionally, it is complexed with ATP. The coupled opening and closure of the NBDs interface has been described as dependent on rigid-body rotations of the TMDs^27^. Since MsmX was crystallized in the absence of ligands, the structure here described very likely corresponds to the open conformation of this multitask NBD protein that should form a dimeric structure upon ATP binding.

**Figure 7:**
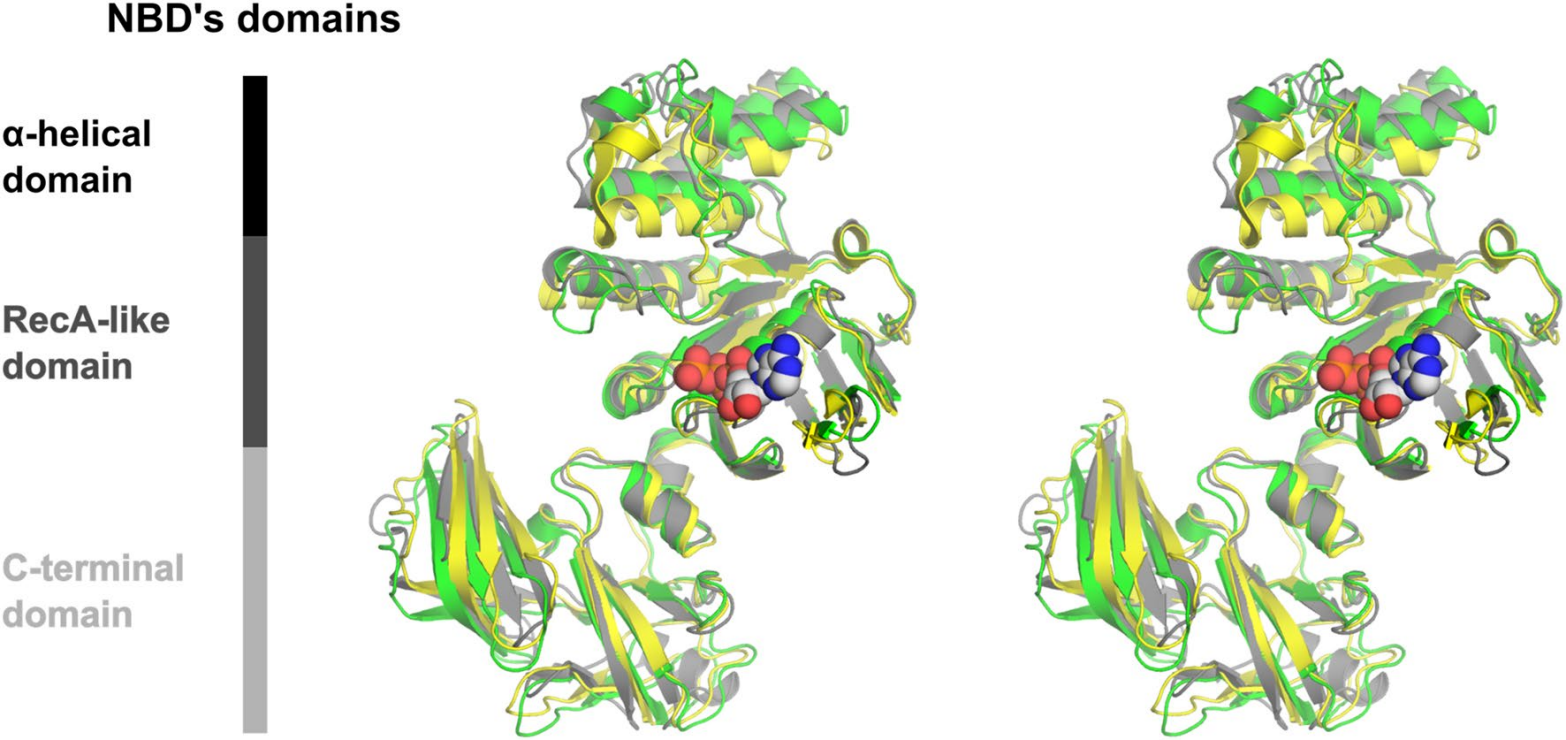
Structural superposition of MsmX K43A and MalK in the ligand-free and complexed with ATP forms. Stereo view of the cartoon representation of the superposition of MsmX K43A (grey), ligand-free MalK (green) (PDB ID 1Q1E:A) and ATP-bound MalK (yellow) (PDB ID 1Q12:A). The ATP ligand from PDB ID 1Q12 is represented as space filling mode. Figure obtained with The PyMOL Molecular Graphics System, Version 2.0.5 Schrodinger, LLC (https://pymol.org/2/).

The overall RMSD between *B. subtilis* MsmX and *E. coli* MalK in its ligand-free form (PDB ID 1Q1E) is 1.84 Å for 337 superimposed C_α_ atoms (Supplementary Table 3). However, the superposition of both structures clearly shows higher structural conservation at the N-terminal domains of both proteins in comparison to the C-terminal domains (Fig. 7): the RMSD value is 1.41 Å for the superposition of 221 C_α_ atoms of the N-terminal domain, and 2.02 Å for 115 C_α_ atoms of the C-terminal domain. This result is not surprising, since the C-terminal domain of NBDs is involved in regulatory roles, while the N-terminal one is mostly involved in the catalytic activity and interaction with TMDs, and hence, more conserved^8^ (see below).

**Figure 8:**
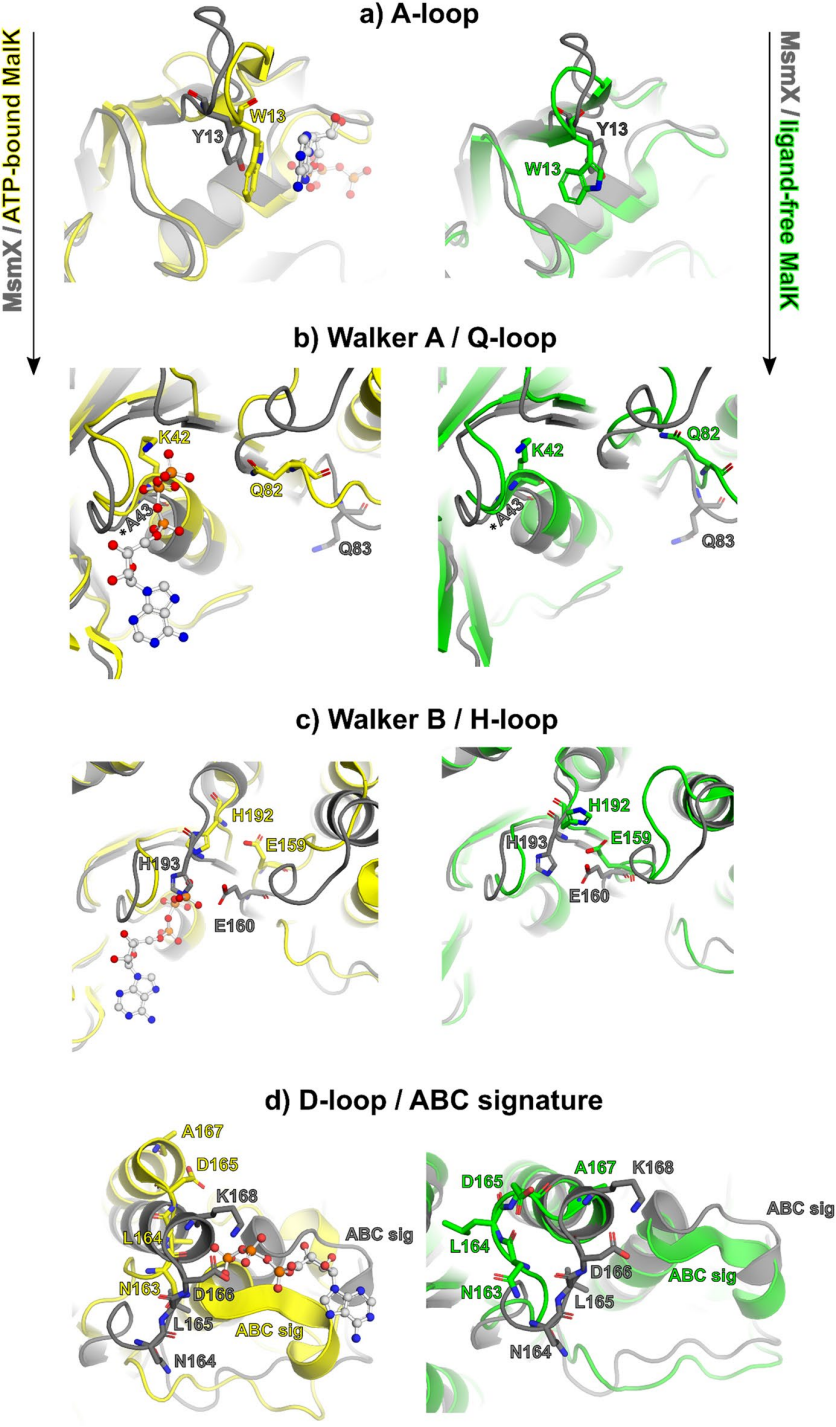
Structural characterization of MsmX functional motifs. Representation of NBDs functional motifs of the structural superposition of MsmX K43A (grey), ligand-free MalK (green) (PDB ID 1Q1E:A) and ATP-bound MalK (yellow) (PDB ID 1Q12:A): A-loop (**a**), Walker A and Q-loop (**b**), Walker B and H-loop (**c**), D-loop and ABC signature (**d**). The most important molecular determinants in each functional motif are represented as sticks and labelled with the color correspondent to the structure. The ATP ligand from PDB ID 1Q1E is represented as ball and stick. Figures obtained with The PyMOL Molecular Graphics System, Version 2.0.5 Schrodinger, LLC (https://pymol.org/2/).

The functional motifs conserved within NBD proteins are present exclusively in the N-terminal domain and were assigned based on *E. coli* MalK protein structures in its ligand-free (PDB ID 1Q1E) and ATP-bound forms (PDB ID 1Q12) (Figs. 5, 6, 7). The A-loop (residues 11–19), located between the antiparallel β1 and β2 strands of NBDs, is the least conserved motif between the two proteins, with different conformations depending on the presence or absence of ATP (Fig. 8a). In the case of MalK, the β-strands are much longer in the ligand-free form than in the ATP-bound form, suggesting that substrate binding gives rise to a longer and more flexible loop. In the ligand-free MsmX K43A structure here reported, the A-loop length is similar to the one in ATP-bound MalK, already in a conformation that favours nucleotide binding. Also contributing to nucleotide accommodation is the hydrophobic residue Y13, present in the A-loop and responsible for ATP positioning: in MalK, it is W13 located next to β1 strand; in MsmX the corresponding Y13 is located in the middle of the A-loop, possibly conferring higher degree of flexibility to bind ATP (Fig. 8a). The Walker A motif (residues 37–44) is fully conserved in MsmX K43A and MalK (Figs. 6 and 8b), and, as expected, the MsmX A43 residue superposes with MalK K42. In the MsmX Q-loop (residues 81–87), Q83 structurally aligns with the conserved glutamine (Q82) involved in the hydrolysis cycle of ATP in the ligand-free and ATP-bound MalK structures (Fig. 8b).

Walker B comprises residues 155–160, which form strand β7. The sequence is conserved in MsmX and MalK (Fig. 6), except for L157 of MalK that is replaced by M158 in MsmX. This small difference must not affect significantly the interaction with strand β8 since the hydrophobic nature of the residue is maintained. Walker B MalK D158 and E159, responsible for Mg^2+^ coordination and water polarization during ATP hydrolysis, respectively, are in the same position as the corresponding residues in MsmX (D159 and E160)^8^. The H-loop (residues 192–194) is well conserved between MsmX and MalK, both at the sequence (Fig. 6) and structural levels (Fig. 8c), suggesting that MsmX H193 might be involved in the interaction with the γ-phosphate of the ATP substrate. In our MsmX K43A structure we observe the H193 imidazole ring is occupying the ATP site, at 4 Å from a sulphate anion, probably from the crystallization solution. The sulphate anion atoms are at the same position as the α-phosphate of the ATP molecule in the MalK ATP-bound form.

The D-loop (residues 163–166) is located downstream of Walker B (Fig. 6). The structural alignment of the two proteins, however, shows that MsmX D-loop and the neighbouring residues (160–170) superpose poorly with MalK either in the ATP-bound or in the ligand-free forms (Fig. 8d). MsmX D166 is at 7.3 Å from the corresponding residue of MalK ligand free, and at 8.9 Å from ATP-bound form, occupying the ATP binding site. N163, L164 and D165 side chains of ligand-free MalK are facing the dimerization interface, while in MsmX, these are pointing inwards (Fig. 8d). Next to the D-loop, K168 of MsmX affects the electrostatic potential of this region, that is more hydrophobic in MalK (with A167 in the corresponding position). Together, these differences suggest that this region is likely to go through conformational changes in order to allow NBD dimerization and substrate binding. The ABC signature (ABC motif in Fig. 6, residues 135–139), characteristic of the ABC superfamily of P-loop NTPases^8^, is strictly conserved between MsmX and MalK protein sequences (Fig. 6). In MsmX crystal structure, this motif is located between α-helices 5 and 6, while in MalK, this region forms a single α-helix and has a 4.7 Å shift upon ATP binding (Fig. 8d).

The regulatory C-terminal domain is the most diverse region within NBDs of ABC transporters, since it is strictly related with regulatory mechanisms, such as carbohydrate preference (Fig. 7)^32^. At secondary structure level, MsmX presents a unique helix α11, between β13 and β14, while *E. coli* MalK possesses an extended loop in this region. The secondary structure assignment of both MsmX and MalK (PDB ID 1Q1E) proteins was confirmed by Promotif^33^ using the PDBsum server^34^ and STRIDE software^35^.

## Discussion

Until two decades ago, it was generally accepted that the component domains of ABC transporters were dedicated to only one specific transporter complex^36^. However, the advent of bacterial genome sequencing led to the suggestion that a single ATPase protein can serve several ABC transporters based on indirect bioinformatic evidence^12,37^. Later, our laboratory and others demonstrated that Gram-positive bacteria have orphan NBDs that are able to energize multiple ABC type I sugar importers^14,15,17^. Here, we show that these two characterized multitask ATPases, MsmX from *B. subtilis* and MsmK from *Strep. pneumoniae,* are functionally exchangeable (Fig. 2 and Supplementary Table 1). Interspecies functional exchangeability was also observed with other Gram-positive pathogens belonging to the Firmicutes phylum: the insect pathogen *B. thuringiensis* serovar *kurstaki*, the human pathogens *C. difficile* and *Staph. aureus*. Hybrid AraNPQ and GanSPQ transporters display distinct efficiency to transport their specific substrates in the host *B. subtilis*, as evaluated by the ability to support growth in the presence of the substrates as sole carbon and energy source (Fig. 2 and Supplementary Table 1). These three NBDs are classified as putative NBDs in the respective annotated genomes and the first two, from *B. thuringiensis* and *C. difficile*, exist as orphan ATPases because no other components of ABC transporters are encoded in their vicinity (Supplementary Fig. 1). On the other hand, the less efficient energy-coupling component in the assembled hybrid transporters, the NBD from *Staph. aureus* appears to belong to an operon encoding a complete ABC carbohydrate uptake system (Supplementary Fig. 1).

Intra-species exchangeability was previously observed between FrlP (YurJ) from *B. subtilis* and MsmX in the hybrid transporter AraNPQ when the gene *frlP (yurJ)* was ectopically expressed^15^. Here we show that FrlP (YurJ), like MsmX, is also able to energize the GanSPQ transporter (Fig. 2 and Supplementary Table 1). FrlP (YurJ) is encoded by a gene adjacent to an operon comprising other components of a system for uptake of fructosamines^38,39^. Functional exchangeability of two distinct and apparently specific NBDs, encoded in operons together, or adjacent, to the other components of a complete ABC type I, have been described before^40^. The UgpC and MalK of *Escherichia coli*, are NBDs of ABC type I transporters comprised in operons with their cognate TMDs and SBP, transporting *sn*-glycerol-3-phosphate and maltose, respectively. Using genetic complementation experiments UgpC and MalK were shown to be functionally exchangeable but the hybrid transporters were less efficient than the wild-type^40^. Similarly, two ABC ATPases of *Streptococcus mutans*, MsmK and MalK, encoded by genes located in the vicinity of the remaining components of their partner ABC transporter, can substitute each other and energize both transporters for raffinose/stachyose and for maltodextrins, respectively^41^. Our results reinforce the observation that functional exchangeability of NBDs has been exclusively reported in bacterial ABC sugar importers of the subfamily CUT1 that transport di‐, tri‐ and higher oligosaccharides. Sharing the ATPase may allow for additional levels of regulation over the ABC sugar importers. *B. subtilis* MsmX has a similar C-terminal domain to MalK from *E. coli*, which has been shown to contribute to carbohydrate preference^32^, and thus a similar role is plausible.

**Figure 9:**
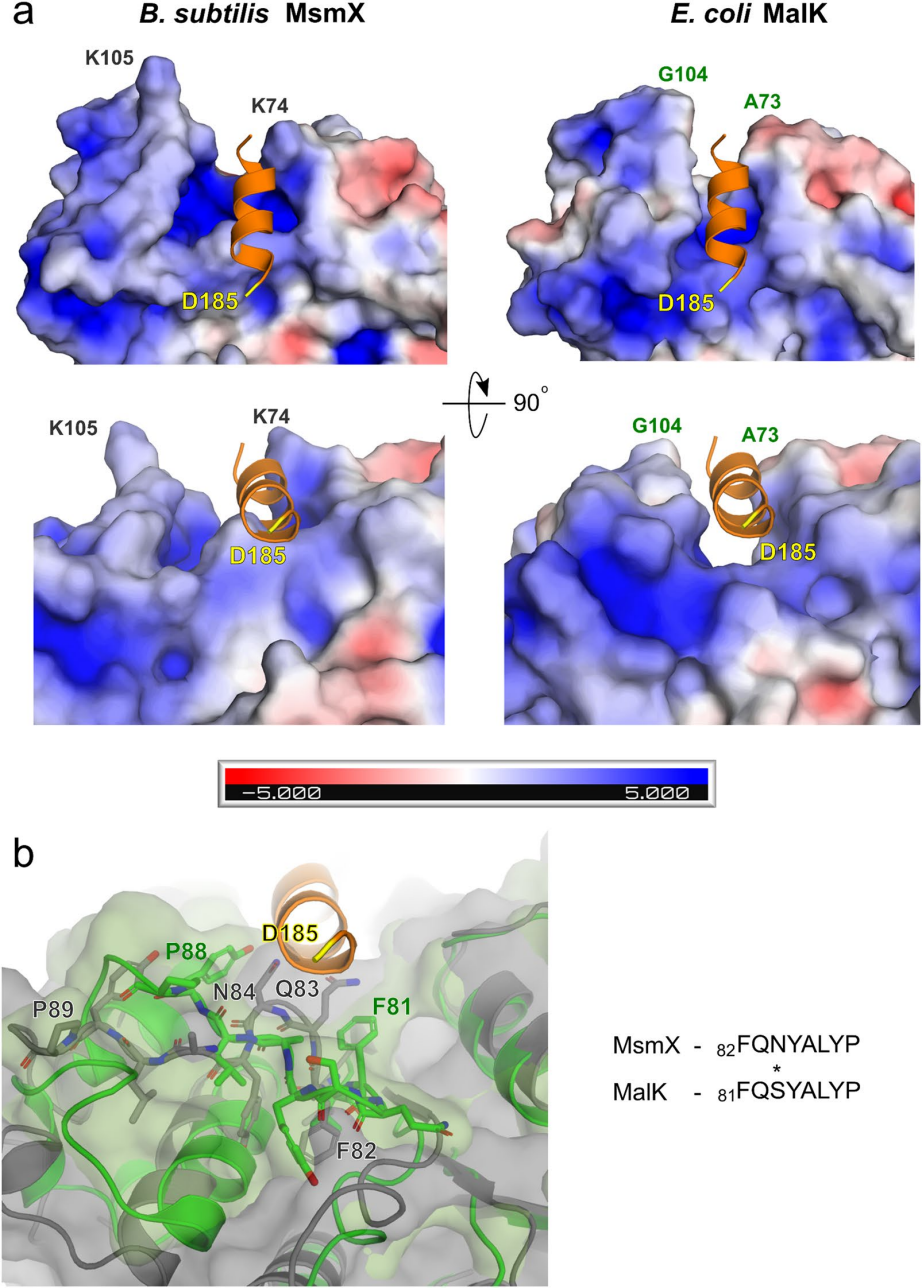
MsmX electrostatic potential at the putative interaction site with TMDs. (**a**) MsmX and MalK electrostatic potential at the NBD-TMD interface, based on MalFGK_2_ complex (PDB ID 3FH6:A). Top and lower panels correspond to a rotation of 90°. The ‘EAA’ helix from MalG protein (PDB ID 3FH6:G) is coloured orange, and its N-terminal residue (D185) is coloured yellow as reference for inter-figure interpretation. The panels on the right show the location of the helix in the MalFGK_2_ complex, while the ones on the left show that MsmX-TMD complex formation requires structural rearrangements or different conformations, otherwise the residues at the surface of MsmX would clash with the corresponding TMD helix. The potential isocontours are shown at + 5 κT/e (blue) and -5 κT/e (red). (**b**) Structural superposition of MsmX at the hydrophobic pocket of MalK (PDB ID 3FH6:A), responsible to accommodate MalG (PDB ID 3FH6:G). Residues forming the pocket are shown as sticks and their sequence alignment is provided. MsmX and MalK structures and respective surfaces are colored grey and green, respectively. Figures obtained with The PyMOL Molecular Graphics System, Version 2.0.5 Schrodinger, LLC (https://pymol.org/2/).

In order to identify potential signature motifs related with the multitasking role of this family of proteins, we analyzed the crystal structure of MalK, inspecting the residues involved in the interface with the MalF and MalG proteins. Since this interface is conserved among the different catalytic conformations of the MalFGK_2_ complex during maltose transport, we used a MalFGK_2_ structure (PDB ID 3FH6:A) with the MalK dimer in the open conformation and no ligands bound^27^, for the comparison with MsmX K43A crystal structure (RMSD of 2.12 Å for 310 C_α_ atoms superposed). The ‘EAA’ motif^42^ of each TMD contains the conserved coupling helix^43^ that interacts with MalK through a surface cleft involving the Q-loop and a hydrophobic pocket. The MalK F81-Y87 region accommodates the MalF/G helix, providing an hydrophobic environment; in MsmX, the corresponding phenylalanine (F82) is flipped, causing the side chains of Q83 and N84 to point towards the complex interface, which decreases the hydrophobic character of the cleft (Fig. 9a and b). Interestingly, N84 is conserved in all NBDs capable of substituting MsmX energizing function, while MalK possesses a serine residue at this position (S83) (Fig. 3a); this difference might affect the range of possible interactions with the coupling helix of TMDs. Furthermore, in the hydrophobic pocket involved at the interface with the MalF/G, MalK A73 is at 3 Å from the MalF helix, while in MsmX this residue is replaced by K74, which affects considerably the electrostatic potential, the volume and the hydrophobicity of the corresponding pocket (Fig. 9a). The diverse features of the MsmX pocket, in particular its larger surface area, may potentially contribute to the interaction with multiple TMDs.

**Figure 10:**
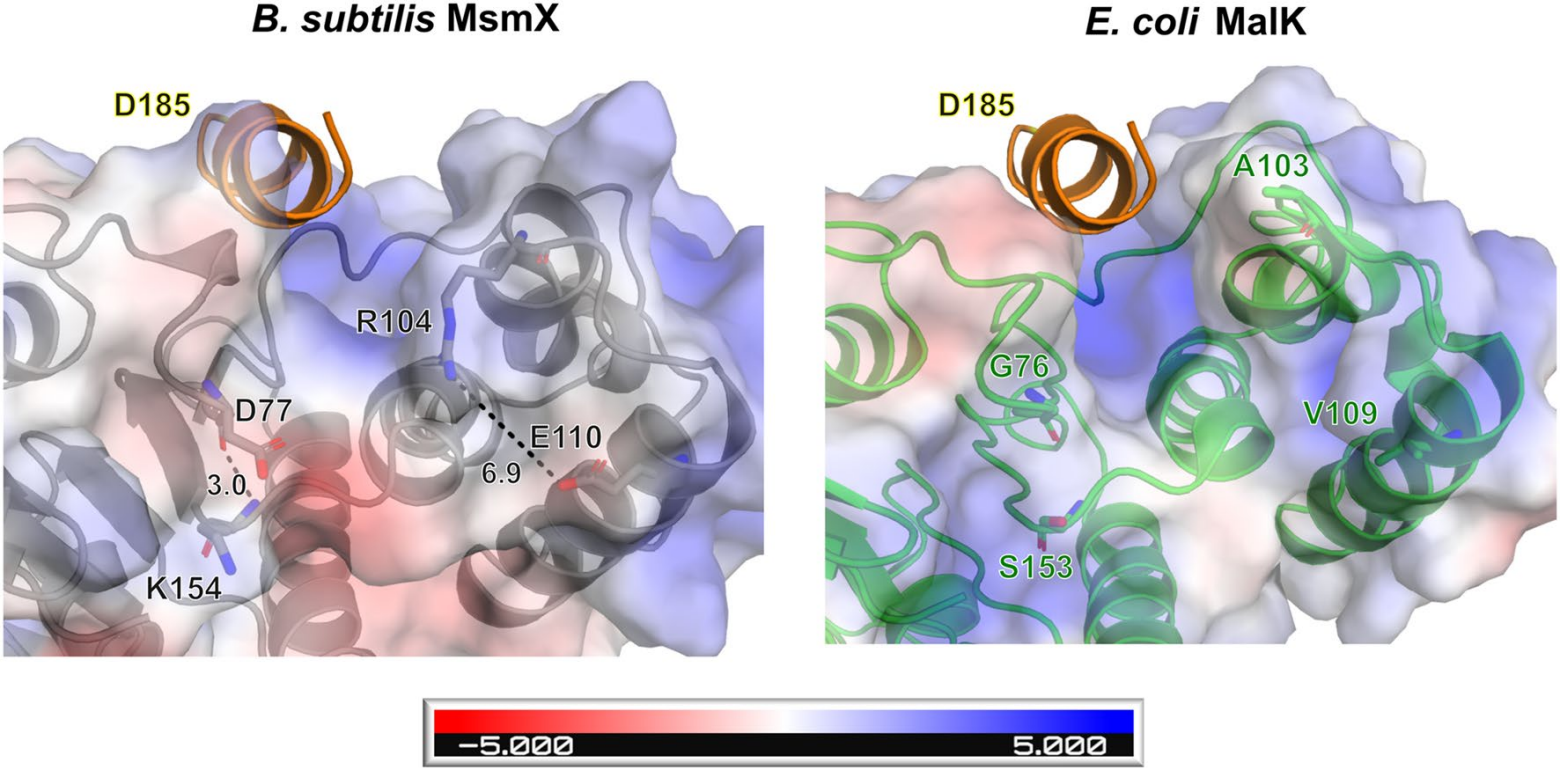
Putative role of surface electrostatic potential for MsmX multitask function. Representation of the electrostatic potential for MsmX and MalK involving conserved residues of multitask NBDs. Relevant MsmX amino acids and the correspondent MalK residues are represented as sticks and distances in Å. MalG ‘EAA’ helix, from MalFGK_2_ complex (PDB ID 3FH6:G) is in orange. The panels on the left shows the putative location of the TMD interaction site. MsmX and MalK (PDB ID 3FH6:A) from MalFGK_2_ are shown as cartoon and colored gray and green, respectively. The potential isocontours are shown at + 5 κT/e (blue) and -5 κT/e (red). Figures obtained with The PyMOL Molecular Graphics System, Version 2.0.5 Schrodinger, LLC (https://pymol.org/2/).

The sequence alignment presented in Fig. 3a shows that the charged residues D77, R104, E110 and K154, conserved among NBDs able to functionally replace MsmX, are exchanged by hydrophobic or neutral residues in *E. coli* MalK (G76, A103, V109 and S153) and YcjV. The structural comparison of MsmX K43A with MalFGK_2_ indicates that these residues, although not located at the complex interface with the TMDs, may contribute to a charged environment in all the multitask proteins (Fig. 10). D77 and K154 in the MsmX K43A structure are not forming a salt bridge but are H-bonded through the main chain atoms (3 Å between the peptide carbonyl of D77 and NH of K154). These two residues are located just upstream of strands β6 and β7, respectively, which flank the α-helical domain (Fig. 6). In addition, D77 might have an additional role considering its proximity to the hydrophobic pocket residues. This analysis supports the observation that D77A and D77A/K154A variants had a pronounced effect in impairing cell growth and sugar transport energization (Fig. 3b). Given the role of the helical domain in the NBD-TMD interaction in ABC transporters^8^, residues D77 and K154 may be involved in conformational rearrangements involving salt bridges/H-bonds needed to select diverse TMDs for complex assembling. R104 and E110 also belong to the α-helical domain of MsmX, with R104 sitting in a loop between α3 and α4, and E110 part of α4 (Fig. 6). The two charged residues (R104, E110) in MsmX K43A are not interacting (nearest distance of the side chains is 6.9 Å) but again are contributing to the charged environment of this region, compared to MalK (Fig. 10). In addition to the putative role of the mutated residues in NBD-TMD interaction, the possibility of their involvement in the correct conformation of the assembled transporter, required to drive the translocation of the substrate across the membrane, should also be considered.

Overall, the structural superposition of MsmX and MalFGK_2_ at the NBD-TMD interface is poor, suggesting some degree of flexibility to account for putative rearrangements upon complex formation. Additionally, all targeted residues may influence the electrostatic potential of this region in the multitask ATPases, with a putative role in the promiscuous interaction with TMDs from different transporters. Interestingly, ABC_Syn from *Synechocystis sp.* complemented MsmX in vivo, but when compared to MsmX, D77 and E110 are conserved in the cyanobacteria protein, while R104 and K154 are replaced by Q103 and S153 (ABC_Syn numbering) (Fig. 3a). These observations support that multitasking activity is not dependent on individual mutations but on the overall structure and electrostatic potential of the interaction region that might vary between phylum. Further structural investigation of these multitask ATPases in the context of the multisubunit complex transporter will clarify with greater detail the sequence and structural signatures associated with the multitask role of these ATPases.

Over the last decade, multitask ATPases have been shown to play a central role in carbohydrate uptake in Firmicutes and its inactivation reduces colonization and attenuates virulence in pathogenic species^15,17,44^ . To our knowledge this study is the first to show that these NBDs of ABC type I importers are functionally exchangeable between species among the Firmicutes. The results provide evidences that multitask ATPases are present in other bacteria phyla and may be widespread in bacteria. Furthermore, the first high resolution crystal structure of an assigned multitask NBD provides a framework to understand the basis of the broader specificity of interaction with the TMDs. It remains to be seen if ABC transporters could be targets for antibacterial therapy development^45,46^ but these specific multitask ATPases, which are not present in humans, may represent a new target to disrupt carbohydrate uptake.

## Methods

### DNA manipulation and sequencing

DNA manipulations were carried out as described previously by Sambrook et al.^47^. Restriction enzymes, T4 ligase and FastAP Thermosensitive Alkaline Phosphatase used in DNA cloning were purchased from Thermo Fisher Scientific and were used in accordance with the manufacturer’s instructions. DNA ligations were performed using T4 DNA Ligase. For PCR reactions Phusion High-Fidelity DNA Polymerase, also from Thermo Fisher Scientific, was used. Clean-up of DNA from agarose gels and restriction and PCR reactions was performed with GFX Gel Band Purification kit (GE Healthcare Life Sciences). Plasmid DNA extraction and clean-up was done using the NZYMiniprep kit from NZYTech. Plasmid DNA and PCR product Sanger sequencing was performed at STAB VIDA (https://www.stabvida.com/). All primers used in this work are listed in Supplementary Table 4.

### Construction of vectors for expression of wild-type and mutagenized MsmX in *E. coli*

All vectors used in this work are described in Supplementary Table 5. pMJ22^15^ was used as a vector for the expression of wild-type MsmX in *E. coli* BL21(DE3)pLysS. Mutagenic primers ARA757 and ARA758 introduced a mutation in codon AAA (Lys) to GCA (Ala), substituting the lysine at position 43 of MsmX for an alanine, creating the expression vector pAM11.

### Construction of vectors for the expression of wild-type and mutagenized MsmX alleles and homolog proteins in *B. subtilis*

Vector pAM13 is a derivative of pAM4 harboring the K43A mutation. Expression vector pPS7 was obtained by amplification of a DNA fragment from pSN74 (a derivative of pDR111, I. Sá-Nogueira, unpublished results) containing the *lacI* gene and the terminal end of the *msmX* gene with oligonucleotides ARA854 (mutagenic primer containing a unique restriction site for *Bgl*II and two novel codons) and ARA632; two new amino acids (Leu, Glu) were introduced into the *msmX* coding region preceding the C-terminal His_6_-tag. The resulting amplification product was digested with *Bgl*II and *Bam*HI and the product was sub-cloned into the same sites of pSN74, yielding pPS7. Vectors for the ectopic expression of either mutagenized *msmX* and other homologs were constructed by amplification of each gene of interest with primers harboring unique restriction sites for *Nhe*I and *Bgl*II and subsequent cloning in the same sites of vector pPS7.

### Construction of *B. subtilis* strains

The ectopic regulated expression of *msmX*, its mutated alleles or homologs, was carried out by placing each gene under the control of an IPTG (isopropyl β-D-1-thiogalactopyranoside)-inducible promoter using the vector pPS7 (Supplementary Table 5) and subsequent integration at the *amyE* locus of the *B. subtilis* chromosome of strain IQB495 (Δ*msmX*::*cat*) by a double recombination event. Transformation of *B. subtilis* was carried out based on the protocol by Anagnostopoulos and Spizizen, 1960^48^. All strains constructed and used in this work are listed in Supplementary Table 6.

### Growth conditions

*E. coli* XL1-Blue or *E. coli* XL10-Gold (Stratagene) were used for the construction of all plasmids. All *E. coli* strains were grown in liquid Lysogeny Broth (LB) medium^49^ and on LB solidified with 1.6% (w/v) agar, where ampicillin (100 μg/mL), chloramphenicol (25 μg/mL), or tetracycline (12 μg/mL) were added as appropriate. *B. subtilis* strains were grown in liquid LB medium, LB solidified with 1.6% (w/v) agar or liquid SP medium^50^, and chloramphenicol (5 μg/mL), spectinomycin (60 μg/mL), or IPTG (1 mM) were added as appropriate. Growth kinetics parameters of *B. subtilis* strains were determined in CSK minimal medium in the presence of 0.1% (w/v) of α- 1,5-arabinotriose or galactan (Megazyme), as described by Ferreira and Sá-Nogueira^14,15^. The optical density (OD_600nm_) of growing cultures was used for the determination of the doubling time for each strain in the presence of the tested sugars as sole carbon and energy sources.

### Statistical analysis

Statistical differences in doubling time were confirmed by the Student’s *t* test for paired samples, when compared with the *B. subtilis* strain ISN10 (*msmX*). FDR-corrected *p*-value (FDR *p*) of 0.05 was used as a cutoff for significance. Statistical analysis was performed using the R software^51^.

### Protein extracts of *B. subtilis* and Western Blot analysis

*B. subtilis* strains were grown in CSK minimal medium supplemented with 0.1% (w/v) of arabinose, as previously described for growth kinetics parameters and doubling time determination^14^. Total protein content and its quantification were performed as described previously^15^. 20 μg of total protein from each extract and 0.25 μg of purified MsmX-His_6_ were loaded in a 12.5% SDS-PAGE handcast gel and run at constant electrical current (30 mA) for 50 min. Transferred proteins were visualized in the membranes with Ponceau Red. The membranes were blocked with powdered milk solution in TBS-Tween (5% w/v), washed and then blotted overnight with a mix of anti-His 1:2,000 (6xHis Epitope TAG, PA1-983B, Thermo Fisher Scientific) and anti-flagellin 1:10,000 (polyclonal antibody raised in rabbit—a gift from Paulo Tavares, I2BC, Gif-sur-Yvette), followed by incubation with HRP-conjugated goat anti-rabbit IgG antibody 1:10,000 (Thermo Scientific, Pierce Antibody Products). Flagellin detection constitutes the loading control. Signal was detected by enhanced chemiluminescence using SuperSignal West Pico PLUS (Thermo Fisher Scientific); Amersham Hyperfilm plates (GE Healthcare Life Sciences) were exposed to luminescence inside a closed Hypercassette Autoradiography Cassette (GE Healthcare Life Sciences).

### Accession number of homolog proteins of MsmX and sequence alignment

The accession numbers of homolog proteins are available at NCBI and can be found in the following links: FrlP (*B. subtilis*) https://www.ncbi.nlm.nih.gov/protein/NP_391135.1; ABC_Bt (*B. thuringiensis*) https://www.ncbi.nlm.nih.gov/protein/WP_000818931.1; ABC_Sa (UgpC) (*S. aureus*) https://www.ncbi.nlm.nih.gov/protein/WP_000818906.1; ABC_Cd (*C. difficile*) https://www.ncbi.nlm.nih.gov/protein/WP_011861561.1; YcjV (*E. coli*): https://www.ncbi.nlm.nih.gov/protein/WP_000057985.1; ABC_Syn (*Synechocystis* sp.) https://www.ncbi.nlm.nih.gov/protein/WP_010874222.1.

Sequence alignments were performed using the JalView version 2 software^52^ or EMBOSS Needle^53^.

### Construction of vectors for expression of wild-type and mutagenized MsmX in *E. coli*

pMJ22^15^ was used as a vector for the expression of wild-type MsmX in *E. coli* BL21 DE3(pLysS). Mutagenic primers ARA757 and ARA758 introduced a mutation in codon AAA (Lys) to GCA (Ala), substituting the lysine at position 43 of MsmX for an alanine, creating the expression vector pAM11.

### Protein expression and purification

MsmX wild-type protein and the K43A mutant from pMJ22 and pAM11 vectors, respectively, were heterologously expressed in *E. coli* BL21(DE3)pLysS cells. Cells were grown aerobically in LB medium at 37 °C and protein expression was induced during exponential cellular growth phase with 0.1 mM IPTG during 16 h at 16 °C. Cells were harvested by centrifugation at 9000 × *g* for 15 min and pellets readily used for purification or stored at—80 °C.

Frozen or fresh pellets were resuspended in buffer A (10 mM PBS pH 7.4, 500 mM NaCl, 10% glycerol and 10 mM Imidazole) supplemented with 10 μg/mL DNaseI and 10 mM MgCl_2_. Cell lysis was performed by sonication and the resulting supernatant applied onto a 5 mL HisTrap HP Column (GE Healthcare Life Sciences). Proteins retained in the column were eluted using a linear gradient of imidazole. Fractions containing MsmX protein were pooled and desalted to buffer B (10 mM Tris–HCl pH 7.4 @ 20 °C, 300 mM NaCl, 10 mM MgCl_2_) using 5 mL HiTrap Desalting columns (GE Healthcare Life Sciences). Protein fractions were pooled and concentrated with Vivaspin Turbo concentrators with 10 kDa molecular weight cut-off (Sartorius), and the resulting sample applied onto a Superdex 75 10/300 GL (GE Healthcare Life Sciences) pre-equilibrated with buffer B. In the case of MsmX wild-type, purification was optimized by including in the buffer A benzonase 5 mU/mL (instead of DNAseI) and ½ tablet of cOmplete ULTRA Tablets, Mini, EDTA-free, EASYpack Protease Inhibitor Cocktail (Roche); and using buffer B with slightly different composition (50 mM Tris–HCl pH 6.8 @ 20 °C, 300 mM NaCl, 10 mM MgCl_2_, 10% glycerol and 5 mM 2-mercaptoethanol). The final yield of MsmX wild-type and K43A mutant was 20 and 40 mg/L of culture, respectively.

### Crystallization, data collection and structure determination of MsmX K43A

Crystallization of wild-type MsmX, in spite of extensive attempts resulted only in poorly diffracting crystals. Therefore, further crystallization trials were pursued with MsmX K43A variant. These were performed at 20 °C with the crystallization robot Oryx 8 (Douglas Instruments Limited) using the sitting drop vapour-diffusion method, with 0.5 μL of protein at 30 mg/mL and 0.5 μL of reservoir solution containing 100 mM (NH_4_)_2_SO_4_, 100 mM HEPES pH 7.5 and 30% (w/v) polyethylene glycol 400 (from the MemStart + MemSys HT-96 screen, MD1-33, Molecular Dimensions). Crystals with maximum dimensions of 0.1 mm × 0.3 mm × 0.06 mm were formed within four days. The diffracting properties of the MsmX K43A mutant crystals decreased during handling, harvesting and cryoprotection with different solutions. The best diffracting data was obtained when the crystals were flash cooled in liquid nitrogen directly from the crystallization drop, taking advantage of the cryoprotectant properties of polyethylene glycol 400.

A complete dataset was collected from a crystal diffracting up to 1.67 Å on ID23 beamline of the European Synchrotron Radiation Facility (ESRF, Grenoble, France). X-ray diffraction data reduction was processed using the software package *XDS*^54^. Anisotropic data correction and cut-off of merged intensities were performed using STARANISO web server^55^ followed by scaling with *Aimless*^56^ software from CCP4^57^program suite. The number of molecules per asymmetric unit was estimated based on Matthews coefficient^58^. Structure determination was achieved by molecular replacement using *Phaser* software^59^ and an ensemble of structures—PDB ID 1V43 and 1Q12—as a search model; the N-terminal and C-terminal domains were searched separately to achieve a partial and a final solution, respectively. Arp/wARP^60^ was used for partial model building with subsequent manual inspection using Coot^61^. Structure refinement was performed interactively using REFMAC5^62^ from CCP4 suite, Phenix.refine^63^ and Coot. Final R_work_ and R_free_ values are 0.20 and 0.25, respectively. The quality of the model was assessed by MolProbity^64^. Statistics of data collection and structure refinement are shown in Supplementary Table 7. The model coordinates and structural factors were deposited in PDB under the accession code 6YIR. Secondary structure elements were assigned using STRIDE software^35^ and protein surface electrostatics calculated with APBS software^65^.

## Supplementary information

Supplementary Information.

## References

[1] Higgins CF (1992). ABC transporters: from microorganisms to man. Annu. Rev. Cell Biol.

[2] Swier, L. J. Y. M., Slotboom, D. J. & Poolman, B. ABC importers: A structure-function perspective. in *ABC Transporters - 40 Years on.* 3–36 (Springer International Publishing, Berlin, 2015).

[3] Higgins CF, Linton KJ (2004). The ATP switch model for ABC transporters. Nat. Struct. Mol. Biol..

[4] Locher KP (2016). Mechanistic diversity in ATP-binding cassette (ABC) transporters. Nat. Struct. Mol. Biol..

[5] Shitan N (2003). Involvement of CjMDR1, a plant multidrug-resistance-type ATP-binding cassette protein, in alkaloid transport in Coptis japonica. Proc. Natl Acad. Sci. USA.

[6] Lee M (2008). The ABC transporter AtABCB14 is a malate importer and modulates stomatal response to CO2. Nat. Cell Biol..

[7] Lewinson O, Livnat-Levanon N (2017). Mechanism of action of ABC importers: conservation, divergence, and physiological adaptations. J. Mol. Biol..

[8] ter Beek J, Guskov A, Slotboom DJ (2014). Structural diversity of ABC transporters. J. Gen. Physiol..

[9] Scheepers GH, Lycklama a Nijeholt JA, Poolman B (2016). An updated structural classification of substrate-binding proteins. FEBS Lett..

[10] Mächtel R, Narducci A, Griffith DA, Cordes T, Orelle C (2019). An integrated transport mechanism of the maltose ABC importer. Res. Microbiol..

[11] Rice AJ, Park A, Pinkett HW (2014). Diversity in ABC transporters: type I, II and III importers. Crit. Rev. Biochem. Mol. Biol..

[12] Quentin Y, Fichant G, Denizot F (1999). Inventory, assembly and analysis of *Bacillus subtilis* ABC transport systems. J. Mol. Biol..

[13] Schönert S (2006). Maltose and maltodextrin utilization by *Bacillus subtilis*. J. Bacteriol..

[14] Ferreira MJ, Sá-Nogueira I (2010). A multitask ATPase serving different ABC-type sugar importers in *Bacillus subtilis*. J. Bacteriol..

[15] Ferreira MJ, Mendes AL, Sá-Nogueira I (2017). The MsmX ATPase plays a crucial role in pectin mobilization by *Bacillus subtilis*. PLoS ONE.

[16] Morabbi Heravi K, Watzlawick H, Altenbuchner J (2019). The *melREDCA* operon encodes a utilization system for the raffinose family of oligosaccharides in *Bacillus subtilis*. J. Bacteriol..

[17] Marion C, Aten AE, Woodiga SA, King SJ (2011). Identification of an ATPase, MsmK, which energizes multiple carbohydrate ABC transporters in *Streptococcus pneumoniae*. Infect. Immun..

[18] Linke CM (2013). The ABC transporter encoded at the pneumococcal fructooligosaccharide utilization locus determines the ability to utilize long- and short-chain fructooligosaccharides. J. Bacteriol..

[19] Hurtubise Y, Shareck F, Kluepfel D, Morosoli R (1995). A cellulase/xylanase-negative mutant of *Streptomyces lividans* 1326 defective in cellobiose and xylobiose uptake is mutated in a gene encoding a protein homologous to ATP-binding proteins. Mol. Microbiol..

[20] Tan M-F (2015). MsmK, an ATPase, contributes to utilization of multiple carbohydrates and host colonization of *Streptococcus suis*. PLoS ONE.

[21] Saurin W, Hofnung M, Dassa E (1999). Getting in or out: early segregation between importers and exporters in the evolution of ATP-binding cassette (ABC) transporters. J. Mol. Evol..

[22] Saier MH (2000). Families of transmembrane sugar transport proteins. Mol. Microbiol..

[23] Oldham ML, Khare D, Quiocho FA, Davidson AL, Chen J (2007). Crystal structure of a catalytic intermediate of the maltose transporter. Nature.

[24] Watzlawick H, Heravi KM, Altenbuchner J (2016). Role of the *ganSPQAB* operon in degradation of galactan by *Bacillus subtilis*. J. Bacteriol..

[25] Krissinel E, Henrick K (2007). Inference of macromolecular assemblies from crystalline state. J. Mol. Biol..

[26] Krissinel E, Henrick K (2004). Secondary-structure matching (SSM), a new tool for fast protein structure alignment in three dimensions. Acta Crystallogr..

[27] Khare D, Oldham ML, Orelle C, Davidson AL, Chen J (2009). Alternating access in maltose transporter mediated by rigid-body rotations. Mol. Cell.

[28] Chen J, Lu G, Lin J, Davidson AL, Quiocho FA (2003). A tweezers-like motion of the ATP-binding cassette dimer in an ABC transport cycle. Mol. Cell.

[29] Lu G, Westbrooks JM, Davidson AL, Chen J (2005). ATP hydrolysis is required to reset the ATP-binding cassette dimer into the resting-state conformation. Proc. Natl Acad. Sci. USA.

[30] Oldham ML, Chen J (2011). Crystal structure of the maltose transporter in a pretranslocation intermediate state. Science.

[31] Ose T, Fujie T, Yao M, Watanabe N, Tanaka I (2004). Crystal structure of the ATP-binding cassette of multisugar transporter from *Pyrococcus horikoshii* OT3. Proteins Struct. Funct. Genet..

[32] Bordignon E, Grote M, Schneider E (2010). The maltose ATP-binding cassette transporter in the 21st century—towards a structural dynamic perspective on its mode of action: MicroReview. Mol Microbiol..

[33] Hutchinson EG, Thornton JM (1996). PROMOTIF—a program to identify and analyze structural motifs in proteins. Protein Sci..

[34] Laskowski RA, Jabłońska J, Pravda L, Vařeková RS, Thornton JM (2018). PDBsum: Structural summaries of PDB entries. Protein Sci..

[35] Frishman D, Argos P (1995). Knowledge-based secondary structure assignment. Prot. Struct. Funct. Genet..

[36] Schneider E, Hunke S (1998). ATP-binding-cassette (ABC) transport systems: Functional and structural aspects of the ATP-hydrolyzing subunits/domains. FEMS Microbiol. Rev..

[37] Schneider E (2001). ABC transporters catalyzing carbohydrate uptake. Res. Microbiol..

[38] Deppe VM (2011). Genetic control of Amadori product degradation in *Bacillus subtilis* via regulation of *frlBONMD* expression by FrlR. Appl. Environ. Microbiol..

[39] Nicolas P (2012). Condition-dependent transcriptome architecture in *Bacillus subtilis*. Science.

[40] Hekstra D, Tommassen J (1993). Functional exchangeability of the ABC proteins of the periplasmic binding protein-dependent transport systems ugp and mal of *Escherichia coli*. J. Bacteriol..

[41] Webb AJ, Homer KA, Hosie AHF (2008). Two closely related ABC transporters in *Streptococcus mutans* are involved in disaccharide and/or oligosaccharide uptake. J. Bacteriol..

[42] Saurin W, Koster W, Dassa E (1994). Bacterial binding protein-dependent permeases: characterization of distinctive signatures for functionaily related integral cytoplasmic membrane proteins. Mol. Microbiol..

[43] Hollenstein K, Frei DC, Locher KP (2007). Structure of an ABC transporter in complex with its binding protein. Nature.

[44] Tan MF (2017). The involvement of MsmK in pathogenesis of the *Streptococcus suis* serotype 2. Microbiologyopen.

[45] Garmory HS, Titball RW (2004). ATP-binding cassette transporters are targets for the development of antibacterial vaccines and therapies. Infect. Immun..

[46] Buckwalter CM, King SJ (2012). Pneumococcal carbohydrate transport: Food for thought. Trends Microbiol.

[47] Sambrook J, Fritsch EF, Maniatis T (1989). Molecular Cloning: A Laboratory Manual.

[48] Anagnostopoulos C, Spizizen BYJ (1960). Requirements for transformation in *Bacillus subtilis*. J. Bacteriol..

[49] Miller JH (1972). Experiments In Molecular Genetics.

[50] Martin I, Débarbouillé M, Ferrari E, Klier A, Rapoport G (1987). Characterization of the levanase gene of *Bacillus subtilis* which shows homology to yeast invertase. Mol. Gen. Genet. MGG.

[51] R Foundation for Statistical Computing. R: A language and environment for statistical computing. Vienna, Austria (2017). Available at: www.R-project.org/.

[52] Waterhouse AM, Procter JB, Martin DMA, Clamp M, Barton GJ (2009). Sequence analysis Jalview Version 2-a multiple sequence alignment editor and analysis workbench. Bioinformatics.

[53] Needleman SB, Wunsch CD (1970). A general method applicable to the search for similarities in the amino acid sequence of two proteins. J Mol Biol..

[54] Kabsch W (2010). XDS. Acta Crystallogr..

[55] Tickle, I.J. *et al.* STARANISO. Cambridge, United Kingdom: Global Phasing Ltd (2018). Available at: https://staraniso.globalphasing.org/cgi-bin/staraniso.cgi.

[56] Evans PR, Murshudov GN (2013). How good are my data and what is the resolution?. Acta Crystallogr..

[57] Collaborative Computational Project (1994). The CCP4 suite: Programs for protein crystallography. Acta Crystallogr..

[58] Kantardjieff KA, Rupp B (2003). Matthews coefficient probabilities: Improved estimates for unit cell contents of proteins, DNA, and protein-nucleic acid complex crystals. Protein Sci..

[59] McCoy AJ (2007). Phaser crystallographic software. J. Appl. Crystallogr..

[60] Langer G, Cohen SX, Lamzin VS, Perrakis A (2008). Automated macromolecular model building for X-ray crystallography using ARP/wARP version 7. Nat. Protoc..

[61] Emsley P, Lohkamp B, Scott WG, Cowtan K (2010). Features and development of Coot. Acta Crystallogr..

[62] Murshudov GN (2011). REFMAC5 for the refinement of macromolecular crystal structures. Acta Crystallogr..

[63] Afonine PV (2012). Towards automated crystallographic structure refinement with phenix.refine. Acta Crystallogr..

[64] Williams CJ (2018). MolProbity: More and better reference data for improved all-atom structure validation. Protein Sci..

[65] Baker NA, Sept D, Joseph S, Holst MJ, McCammon JA (2001). Electrostatics of nanosystems: Application to microtubules and the ribosome. Proc. Natl Acad. Sci. USA.

