# Supplementary material for: Multitask ATPases (NBDs) of bacterial ABC importers type I and their interspecies exchangeability
Source: Sci Rep. 2020 Nov 11;10:19564. doi: 10.1038/s41598-020-76444-0 (PMC7658222; doi:10.1038/s41598-020-76444-0)
Supplement: Supplementary file 1 — Supplementary Information. [file 41598_2020_76444_MOESM1_ESM.docx]

**Multitask ATPases (NBDs) of Bacterial ABC importers type I and their Interspecies Exchangeability**

Francisco Leisico ^2^†, Lia M. Godinho^1^†, Inês C. Gonçalves^1^, Sara P. Silva^1^, Bruno Carneiro^2^, Maria J. Romão^2^, Teresa Santos-Silva^2^*, Isabel de Sá-Nogueira^1^*

^1^ Microbial Genetics Laboratory, UCIBIO, Departamento de Ciências da Vida, Faculdade de Ciências e Tecnologia, Universidade NOVA de Lisboa, Quinta da Torre, 2829-516 Caparica, Portugal.

^2^ XTAL – Macromolecular Crystallography Laboratory, UCIBIO, Departamento de Química, Faculdade de Ciências e Tecnologia, Universidade NOVA de Lisboa, Quinta da Torre, 2829-516 Caparica, Portugal.

**Supplementary Information**

**Construction of vectors for expression of mutagenized MsmX K43A in *E. coli***

Expression vector pAM4 was used as template for site-directed mutagenesis using the mutagenic oligonucleotides ARA757 and ARA758. This pair of primers introduced the mutation of codon AAA (Lys at position 43) to GCA (Ala) in the resulting plasmid pAM13. A polymerase chain reaction was carried on using 1x Phusion GC Buffer (Thermo Fisher Scientific), 0.2 μM primers, 200 μM dNTPs, 3% DMSO, 0.6 ng/μL of pMJ22 DNA (or 0.5 ng/μL of pAM4 DNA) and 0.02 U/μL of Phusion High-Fidelity DNA Polymerase in a total volume of 50 μL. The PCR product was digested with 10 U of DpnI, at 37 ºC, overnight. The mutation was confirmed by DNA sequencing.

**Construction of vectors expressing the MsmX homolog proteins**

Amplification of the *ABC_Bt* gene was obtained from chromosomal DNA of strain *Bacillus thuringiensis* serovar *kurstaki* str. HD73 (*Bacillus* Genetic Stock Center, BGSC, Ohio State University) with primers ARA851 and ARA852; primers ARA855 and ARA749 amplified the *msmK* gene from *S. pneumoniae* TIGR4 (a gift from Hermínia de Lencastre – ITQB António Xavier, UNL; *ugpC* (ABC_Sa) from *S. aureus* subsp. *aureus* ST398 (also a gift from Hermínia de Lencastre – ITQB António Xavier, UNL) was amplified with primers ARA843 and ARA844; *B. subtilis* 168T^+^ was used as a template for the amplification of *frlP* with the oligonucleotide pair ARA837 and ARA838; *malK* from *E. coli* K-12 (DSMZ 498) was amplified using the primer pairs ARA847 and ARA848; *ycjV* from *E.coli* K-12 was used as template for site-directed mutagenesis by primer extension (see below), using mutagenic oligonucleotides ARA862 and ARA863 and flanking oligonucleotides ARA860 and ARA861. The pair of mutagenic primers allowed the insertion of a guanidine in codon 320, thereby restoring the wild-type phenotype of the protein YcjV with a total length of 360 amino acids; ARA845 and ARA846 were then used to amplify the *ycjV* gene with unique NheI and BglII sites, respectively. The *ABC_Cd* gene from *C. difficile* strain 630 (a gift from Adriano O. Henriques, – ITQB António Xavier, UNL), encoding a putative ABC transporter was obtained by amplification of genomic DNA with primers ARA883 and ARA884. Finally, the locus encoding for an ABC transporter ATP-binding protein of *Synechocystis* sp PCC6803, *ABC_Syn* (genomic DNA was a gift from Paula Tamagnini, I3S/IBMC) was amplified using primers ARA922 and ARA923. Cloning of MsmX homologs yielded the following plasmids: pPS8 (*ABC_Bt*), pPS9 (*msmK*), pPS10 (*ABC_Sa*), pPS11 (*malK*), pPS12 (*frlP*), pPS14 (*ycjV*), pSN88 (*ABC*_*Cd*) and pIG3 (*ABC_Syn*). All vectors were sequenced to confirm the correct insertion of the homolog proteins. Oligonucleotides and plasmids used in this work are listed in Table 4 and Table 5, respectively.

**Construction of mutagenized alleles of MsmX**

The *msmX* sequence from pPS7 served as template for the mutagenesis of selected residues of MsmX. Mutations were introduced using an overlap PCR extension protocol, using ARA770 and ARA771 as external primers. Asp77 was mutagenized to an alanine (mutation D77A) using internal mutagenic primers ARA892 and ARA900. Two separate PCR reactions were performed to obtain two fragments, one using ARA892 and ARA770 and another using ARA771 and ARA900. The two products were then used as template in an extension PCR reaction. External primers (ARA770 and ARA771) were then added to the reaction to obtain the full-length mutagenized *msmX* gene, which was subsequently subcloned between the BglII and NheI sites of pPS7. Residues arginine 104, glutamate 110 and lysine 154 were mutagenized to alanine (R104A, E110A and K154A) using the same strategy. Mutagenic primers ARA879 and ARA880 introduced the R104A mutation; primers ARA902 and ARA903 mutagenized E110; K154 was mutagenized using internal primers ARA893 and ARA895. Double mutations were inserted using previously mutagenized *msmX* in pPS7 as template (R104A mutant to obtain R104A/E110A and D77A to obtain D77A/K154A). All mutagenized *msmX* alleles were sequenced to confirm the introduction of the mutations.

**Construction of vectors for expression of MsmX, MalK, YcjV and mutagenized MsmX D77A/K154A in *E. coli* BL21 (DE3)**

Primers ARA442 and ARA742 were used for the amplification of *msmX*, *malK*, *ycjV* and *msmX* D77A/K154A using plasmids pPS7, pPS11, pPS14 and pPS7_D77A/K154A as DNA templates, respectively. The *msmX* and *msmX* D77A/K154A DNA fragments digested with HincII and XhoI were subcloned between the EcoRV and XhoI restriction sites of pET30a(+). The *malK* and *ycjV* DNA fragments digested with HindIII and XhoI were subcloned into pET30a(+) restricted with the same enzymes. The resulting plasmids, pIG10 (MalK-LEHis_6_), pIG11 (YcjV-LEHis_6_), pIG12 (MsmX-LEHis_6_) and pIG13 (MsmX D77A/K154A-LEHis_6_) were sequenced to confirm the proper insertion of fragments in the expression vector.

**Western Blot analysis of MsmX-His_6_ in the presence of arabinose and arabinotriose**

20 μg of total protein from each extract and 0.5 μg of purified MsmX-His_6_ were loaded in a 12.5% SDS-PAGE handcast gel and run at constant electrical current (30 mA) for 50 min. Transferred proteins were visualized in the membranes with Ponceau Red. The membranes were blocked with powdered milk solution in TBS-Tween (5% w/v), washed and then blotted overnight with an anti-His 1:1,000 (mouse monoclonal Anti-6X His-tag antibody [HIS.H8; Abcam]) followed by incubation with HRP-conjugated goat anti-mouse IgG antibody 1:10,000 (Jackson ImmunoResearch Europe Ltd.). Signal was detected by enhanced chemiluminescence using SuperSignal West Pico PLUS (Thermo Fisher Scientific). The membrane was then stripped and reprobed with a second primary antibody solution (mouse monoclonal Anti-σ70 [from *E. coli*; a gift from Adriano O. Henriques, – ITQB António Xavier, UNL], diluted 1:1,000, followed by incubation with HRP-conjugated goat anti-rabbit IgG antibody 1:10,000 (Thermo Scientific, Pierce Antibody Products). Signal was detected by enhanced chemiluminescence using SuperSignal West Pico PLUS (Thermo Fisher Scientific); Amersham Hyperfilm plates (GE Healthcare Life Sciences) were exposed to luminescence inside a closed Hypercassette Autoradiography Cassette (GE Healthcare Life Sciences). The results are shown in supplementary Fig. 4.

**Genomic context of putative uncharacterized NBDs**

The genomic organization of each NBD-containing region was retrieved from NCBI Gene Database. Gene context of *frlP*, *ABC_Bt*, *ABC_Sa*, *ABC_Cd*, *ycjV* and *ABC_Syn* can be found using gene IDs 937070, 14558340, 12323215, 4914566, 945890 and 14618496, respectively.

***In vitro* ATPase activity of MsmX and MsmX K43A**

The determination of the *in vitro* ATPase activity of MsmX was assessed by measuring the release of phosphate from ATP hydrolysis through a colorimetric substrate. Using the Malachite Green Phosphate Detection Kit (R&D Systems), free inorganic phosphate complexes with malachite green molybdate at low pH and its amount can be measured by light absorbance at 620 nm. Reactions were performed in a total volume of 600 µL in 50 mM Tris-HCl pH 6.8 @ 20 ^o^C, 300 mM NaCl, 10 mM MgCl_2_, 10% glycerol and 5 mM 2-mercaptoethanol; using 42 µg of purified protein, the reaction was started by adding 0.5 mM ATP. After 4 hours, the amount of free phosphate released from ATP hydrolysis was measured in triplicate according to the kit instructions. ATP Autohydrolysis was subtracted for each set of reactions. KH_2_PO_4_ standard solutions, which were measured in triplicate, were used to generate a linear regression with Abs at 620 nm for free phosphate calibration.

**Expression and purification of recombinant NBDs**

*E. coli* BL21 (DE3) cells harboring pIG10 (MalK-LEHis_6_), pIG11 (YcjV-LEHis_6_), pIG12 (MsmX-LEHis_6_) and pIG13 (MsmX D77A/K154A-LEHis_6_), were grown at 37 ⁰C and 150 rpm in 500 mL of LB with appropriate antibiotic selection. When OD_600nm_ reached 0.6 the expression of the recombinant proteins was induced by the addition of 0.1 mM IPTG and the culture was incubated overnight at 16⁰ C and 150 rpm. Cells were harvested by centrifugation at 4⁰ C (9000 x *g*; 10 min) and cell pellets were stored at - 80⁰ C until further use. All subsequent steps were carried out at 4 ⁰C.

Cell pellets were resuspended in 10 mM PBS pH 7.4, 500 mM NaCl, 10% glycerol, 10 mM Imidazole, 10 mM MgCl_2_ and 5 mM 2-mercaptoethanol. Cell lysis was performed by sonication in the presence of 5 mU/mL benzonase, 1 mg/mL lysozyme and 10 mM phenylmethylsulfonyl fluoride (PMSF) and the soluble protein fraction of cells harboring MalK-LEHis_6_ and MsmX-LEHis_6_ was loaded onto a 1-mL HisTrap column (GE Healthcare Life Sciences). Bound proteins were eluted by discontinuous imidazole gradient, and fractions containing MsmX-LEHis_6_ and MalK-LEHis_6_ were desalted to assay buffer (50 mM Tris-HCl pH 6.8 @ 20 °C, 300 mM NaCl, 10 mM MgCl_2_, 10% glycerol and 5 mM 2-mercaptoethanol) by gel filtration using PD-10 Desalting Columns (GE Healthcare Life Sciences). To obtain recombinant YcjV-LEHis_6_ and MsmX D77A/K154A-LEHis_6_, the insoluble protein fraction of cell cultures was resuspended in 10 mM PBS pH 7.4, 500 mM NaCl, 10% glycerol, 10 mM MgCl_2_ 5 mM 2-mercaptoethanol and 3 M Guanidine HCl and centrifuged at 20 000 x *g* for 1 h. The resulting supernatant was loaded onto a 1-mL HisTrap column (GE Healthcare Life Sciences) and the bound denatured proteins were renatured within the column with buffer 10 mM PBS pH 7.4, 500 mM NaCl, 10% glycerol, 10 mM MgCl_2_ 5 mM 2-mercaptoethanol. Bound proteins were eluted by discontinuous imidazole gradient, and fractions containing YcjV-LEHis_6_ and MsmX D77A/K154A-LEHis_6_ were desalted to assay buffer (50 mM Tris-HCl pH 6.8 @ 20 °C, 300 mM NaCl, 10 mM MgCl_2_, 10% glycerol and 5 mM 2-mercaptoethanol) by gel filtration using PD-10 Desalting Columns (GE Healthcare Life Sciences). MsmX-LEHis_6_ was also prepared and obtained from the insoluble protein fraction for comparative purposes. The analysis of production and molecular mass of the NBDs were determined by sodium dodecyl sulfate-polyacrylamide gel electrophoresis (SDS-PAGE), using the Low Molecular Weight Marker from NZYTech as standard and stained with Coomassie Blue reagent. Protein content was determined using the Bradford reagent (Bio-Rad Laboratories) with bovine serum albumin as the standard.

***In vitro* ATPase activity of recombinant NBDs**

Phosphate release from ATP hydrolysis by MsmX-LEHis_6_ and MalK-LEHis_6_ was assessed through a colorimetric substrate using published protocols ^1^. Briefly, reactions were performed at 20 ^o^C in a total volume of 1000 µL in assay buffer (50 mM Tris-HCl pH 6.8 @ 20 ^o^C, 300 mM NaCl, 10 mM MgCl_2_, 10% glycerol and 5 mM 2-mercaptoethanol) using 140 µg (MsmX-LEHis_6_ and MalK-LEHis_6_) or 100 µg (YcjV-LEHis_6_, MsmX D77A/K154A-LEHis_6_ and MsmX-LEHis_6_) of purified protein, and the reaction was started by adding 0.5 mM ATP. After 4 h, the amount of free phosphate released from ATP hydrolysis was measured in triplicate by light absorbance at 650 nm in two independent assays. ATP autohydrolysis was subtracted for each set of reactions. KH_2_PO_4_ standard solutions, which were measured in triplicate, were used to generate a linear regression with Abs 650 nm for free phosphate calibration.

**Table 1 - Effect of *msmX* deletion and conditional expression of different NBDs on a *msmX*-null background on the uptake of arabinotriose or galactan in *B. subtilis*.** Doubling time (min) of *B. subtilis* mutants in liquid minimal medium (CSK) supplemented with α-1,5-arabinotriose or galactan as sole carbon and energy sources, in the presence or absence of 1 mM IPTG. Results are the average of at least three independent assays and their respective standard deviations. A value above 500 min is considered no growth because this residual growth is due to the presence of mono- and disaccharides in the medium, which are not imported by AraNPQ or GanSPQ^2,5^. ND – not determined.

|  | **Arabinotriose 0.1%** | | **Galactan 0.1%** | |
| --- | --- | --- | --- | --- |
| **Strain/MsmX Homologs** | **no IPTG** | **1 mM IPTG** | **no IPTG** | **1 mM IPTG** |
| IQB495 (Δ*msmX*::*cat*) | 848.8 ± 137.5 | ND | 1,010.0 ± 300.8 | ND |
| ISN10 MsmX (*B. subtilis*) | 525.3 ± 69.3 | 115.4 ± 9.3 | 669.0 ± 144.8 | 172.4 ± 9.3 |
| FrlP (*B. subtilis*) | 706.5 ± 79.8 | 211.5 ± 12.8 | 845.7 ± 577.5 | 390.9 ± 78.2 |
| ABC_Bt (*B. thuringiensis*) | 503.2 ±58.0 | 113.8 ± 6.2 | 618.8 ± 126.3 | 148.6 ± 10.9 |
| MsmK (*Strep. pneumoniae*) | 588.6 ± 50.2 | 153.2 ± 15.8 | 652.2 ± 86.9 | 184.9 ± 13.6 |
| ABC_Cd (*C. difficile*) | 610.8 ± 166.7 | 225.7 ± 9.5 | 786.0 ± 202.8 | 398.2 ± 55.2 |
| ABC_Sa (*Staph. aureus*) | 593.3 ± 53.2 | 373.7 ± 45.8 | 634.3 ± 153.6 | 424.1 ± 68.6 |
| MalK (*E. coli*) | 751.8 ± 110.2 | 805.5 ± 188.4 | 752.6 ± 0.0 | 919.8 ± 144.8 |
| YcjV (*E. coli*) | 562.6 ± 105.6 | 569.0 ± 29.1 | 689.9 ± 125.4 | 652.2 ± 122.9 |
| ABC_Syn (*Synechocystis* sp) | 924.3 ± 240.3 | 328.5 ± 7.4 | 644.0 ± 56.9 | 366.3 ± 69.3 |

**Table 2 - Effect of mutations in MsmX in the uptake of arabinotriose by AraNPQ in *B. subtilis*.** Doubling time (min) of strains bearing MsmX mutant variants in liquid minimal medium (CSK) supplemented with α-1,5-arabinotriose as sole carbon and energy source, in the presence or absence of 1 mM IPTG. Results are the average of at least three independent assays and their respective standard deviations.

| **MsmX mutation(s)** | **no IPTG** | **1 mM IPTG** |
| --- | --- | --- |
| MsmX_D77A | 593.6 ± 131.3 | 180.9 ± 18.7 |
| MsmX_R104A | 522.6 ± 115.6 | 118.4 ± 5.9 |
| MsmX_E110A | 535.3 ± 64.5 | 127.5 ± 8.1 |
| MsmX_K154A | 551.9 ± 57.9 | 142.8 ± 14.6 |
| MsmX_R104A E110A | 520.8 ± 96.3 | 143.7 ± 7.7 |
| MsmX_D77A K154A | 619.9 ± 243.9 | 251.2 ± 17.8 |
| **Strain / relevant genotype** | **no IPTG** | **1 mM IPTG** |
| IQB673 (*msmX* wild-type) | 564.2 ± 123.1 | 107.6 ± 3.9 |
| IQB675 (*msmX* K43A) | 809.9 ± 230.8 | 752.6 ± 250.9 |

**Table 3. The top hits of MsmX homologs derived by PDBeFold**^6^ **search.** The pairwise 3D alignment was performed using the structure of *B. subtilis* MsmX K43A.

| **Protein** | **Organism** | **RMSD (Å)** | **PDB ID:chain** | **Active site ligand** | **Conformation** | **Reference** |  |
| --- | --- | --- | --- | --- | --- | --- | --- |
| MalK | *E. coli* | 1.84 | 1Q1E:A | - | Open | ^7^ |  |
|  |  | 1.70 | 2AWO:A | ADP+Mg^2+^ | Semi-open | ^8^ |  |
|  |  | 2.53 | 1Q12:A | ATP | Closed | ^7^ |  |
| MalFGK_2_ | *E. coli* | 2.12 | 3FH6:A | - | Inward open | ^9^ |  |
| MalFGK_2_-MBP | *E. coli* | 2.50 | 3PV0:A | Maltose | Pre-translocation | ^10^ |  |
|  |  | 2.58 | 2R6G:A | Maltose; ATP | Outward open | ^11^ |  |
| MalK | *P. horikoshii* | 1.70 | 1V43:A | - | Open | ^12^ |  |
|  |  | 1.75 | 1VCI:A | ATP | Closed |  |  |
|  | | | | | | | |

**Table 4. List of oligonucleotides used in this work; restriction sites and mutated nucleotides are underlined.**

| **Oligonucleotide** | **Sequence 5’ 🡪 3’** |
| --- | --- |
| **ARA442** | GAGCTGCCTGCCGCGTTTCGGTG |
| **ARA632** | AGAGCTGTACGCAGCCGCTG |
| **ARA742** | CCCTTGCATGCGGTTTGATTCTGAG |
| **ARA749** | GCACGCATGCACTGATATCTCTCC |
| **ARA757** | TGCGGGGCATCAACGACGCTGCGAATGG |
| **ARA758** | CATTCGCAGCGTCGTTGATGCCCCGCAG |
| **ARA770** | CTTATCGAGATCTCACTTCTGTCTC |
| **ARA771** | TTAGATGGCTAGCTTGCGGATGG |
| **ARA837** | CTGATGGCTAGCTTAACATTTGAACACG |
| **ARA838** | TAAGATCTCTCTTCCGTTTCCGCATCG |
| **ARA843** | GCCAACATGGCTAGCTTAAAGTTAG |
| **ARA844** | GCCGCCAGATCTATTTCCTGTTTTTTC |
| **ARA845** | ATCAACATGGCTAGCCTTTCGTTAC |
| **ARA846** | TTGGCGAGATCTTATTTCCGTTTCTGC |
| **ARA847** | AAGTTTATGGCTAGCGTACAGCTGC |
| **ARA848** | TTAGCCAGATCTCTCCTTATGCAGTCG |
| **ARA851** | GGTACCATGGCTAGCCTTAAATTAG |
| **ARA851** | TTAGCGAGATCTTTGTTCAGTTTGG |
| **ARA852** | TTAGCGAGATCTTTGTTCAGTTTGG |
| **ARA854** | GAAGATCTCGACTCGAGCACCACCATCACCACCACTAAGATC |
| **ARA855** | CGCGCCGTCGACTATATAATATAATTATC |
| **ARA860** | TGGCCAAGCTTATCGGCCTTCTG |
| **ARA861** | CACATCAGGCATGCGGTACAGGG |
| **ARA862** | TTGGGGGGCACGAGTTAGTGG |
| **ARA863** | ACTAACTCGTGCCCCCCAACC |
| **ARA879** | GGCATTTTCGCAAGCTTGAGCCCGAACGC |
| **ARA880** | GCTCAAGCTTGCGAAAATGCCGAAGCC |
| **ARA883** | GGCGCCGGCTAGCGTAATTTTAAAAAATATATCAAAAC |
| **ARA884** | CCGTTAAGATCTAGCTAAAGTAGTTTCTTTG |
| **ARA892** | ACAGGGCTATCGCGATGGTA |
| **ARA893** | AACACCGCTGCATCCCGCA |
| **ARA900** | CATCGCGATAGCCCTGTCCTTTGG |
| **ARA901** | GGGATGCAGCGGTGTTCCTGATGG |
| **ARA902** | CTTTTTTTGATTGCAGGCTTCGG |
| **ARA903** | CCTGCAATCAAAAAAAGAGTCG |
| **ARA922** | CCTATGGCTAGCGTCAGTTTTG |
| **ARA923** | CGAGATCTCGTTTCCATTTCCCGG |

**Table 5 – List of plasmids used in this work.**

| **Plasmid** | **Relevant Construction** | **Source or**  **Reference** |
| --- | --- | --- |
| pDR111 | Derivative of the Pspac(hy) plasmid pJQ43; contains an additional *lacO* binding site | David Rudner |
| pMJ22 | pET30a(+)-based vector for the expression of *msmX*-His_6_, *kan* | ^2^ |
| pAM4 | pDR111 derivate, with *msmX* under the control of Pspank(hy) | ^2^ |
| pAM11 | pMJ22 derivate with *msmX* sequence with mutated AAA (Lys at position 43) to GCA (Ala) | This work |
| pAM13 | pAM4 derivate with *msmX* sequence with mutated AAA (Lys at position 43) to GCA (Ala) | This work |
| pSN74 | pDR111 derivate, with an additional C-terminal His_6_-tag in the coding region of *msmX* under the control of Pspank(hy) | This work |
| pPS7 | pSN74 derivate, with the insertion of two amino acids (L, E) in the *msmX* sequence, followed by the C-terminal His_6_-tag | This work |
| pPS8 | pPS7 derivate, with *ABC_Bt* under the control of Pspank(hy), and with a C-terminal LEHis_6_-tag | This work |
| pPS9 | pPS7 derivate, with *msmK* under the control of Pspank(hy), and with a C-terminal LEHis_6_-tag | This work |
| pPS10 | pPS7 derivate, with *ABC_Sa* under the control of Pspank(hy), and with a C-terminal LEHis_6_-tag | This work |
| pPS11 | pPS7 derivate, with *malK* under the control of Pspank(hy), and with a C-terminal LEHis_6_-tag | This work |
| pPS12 | pPS7 derivate, with *frlP* under the control of Pspank(hy), and with a C-terminal LEHis_6_-tag | This work |
| pPS14 | pPS7 derivate, with *ycjV* under the control of Pspank(hy), and with a C-terminal LEHis_6_-tag | This work |
| pSN88 | pPS7 derivate, with *ABC_Cd* under the control of Pspank(hy), and with a C-terminal LEHis_6_-tag | This work |
| pIG3 | pPS7 derivate, with *ABC_Syn* under the control of Pspank(hy), and with a C-terminal LEHis_6_-tag | This work |
| pPS7_D77A | pPS7 derivative with *msmX* D77 mutagenized to Ala under the control of Pspank(hy), with a C-terminal LEHis_6_-tag | This work |
| pPS7_R104A | pPS7 derivative with *msmX* R104 mutagenized to Ala under the control of Pspank(hy), with a C-terminal LEHis_6_-tag | This work |
| pPS7_E110A | pPS7 derivative with *msmX* E110 mutagenized to Ala under the control of Pspank(hy), with a C-terminal LEHis_6_-tag | This work |
| pPS7_K154A | pPS7 derivative with *msmX* K154 mutagenized to Ala under the control of Pspank(hy), with a C-terminal LEHis_6_-tag | This work |
| pPS7_R104A/E110A | pPS7 derivative with *msmX* R104 and E110 mutagenized to Ala under the control of Pspank(hy), with a C-terminal LEHis_6_-tag | This work |
| pPS7_D77A/K154A | pPS7 derivative with *msmX* D77 and K154 mutagenized to Ala under the control of Pspank(hy), with a C-terminal LEHis_6_-tag | This work |
| pIG10 | pET30a(+)-based vector for the expression of *malK*-LEHis_6_, *kan* | This work |
| pIG11 | pET30a(+)-based vector for the expression of *ycjV*-LEHis_6_, *kan* | This work |
| pIG12 | pET30a(+)-based vector for the expression of *msmX*-LEHis_6_, *kan* | This work |
| pIG13 | pET30a(+)-based vector for the expression of *msmX* D77A/K154A-LEHis_6_, *kan* | This work |

**Table 6 – List of strains used in this work.**

| **Strain** | **Relevant Genotype** | **Source** |
| --- | --- | --- |
| ***E. coli*** |  |  |
| DH5α | *fhuA*2 Δ(*argF*-*lacZ*) *U169* *phoA* *glnV44* Φ80 Δ(l*acZ*)*M15* *gyrA96* *recA1* *relA1* *endA1* *thi-1 hsdR17* | Gibco – BRL |
| XL1Blue | *recA1* *endA1* *gyrA96 thi-1 hsdr17 supE44 relA1 lac* [F’ *proAB lacI*^q^ *Z*Δ*M15* Tn*10* (Tet^r^)] | Stratagene |
| XL10 Gold | Tet^r^Δ (*mcrA*)*183* Δ (*mcrCB*-*hsdSMR*-*mrr*)*173* *endA1* *supE44* *thi-1* *recA1* *gyrA96* *relA1* *lac* Hte [F´ *proAB* *lacI*^q^ZΔ*M15* Tn*10* (Tet^r^) Amy Cam^r^] | Stratagene |
| BL21(DE3) | F- *ompT* *hsdS_B_*(r_B_^-^ m_B_^-^) *gal dcm* (DE3) (Cm^R^) | ^3^ |
| BL21(DE3)pLysS | F- *ompT* *hsdS_B_*(r_B_^-^ m_B_^-^) *gal dcm* (DE3) pLysS (Cm^R^) | ^3^ |
| ***B. subtilis*** |  |  |
| 168T^+^ | Prototroph | ^4^ |
| IQB495 | Δ*msmX*::*cat* | ^5^ |
| IQB673 | Δ*msmX*::*cat* Δ*amyE*::pSpank(hy)-*msmX* | ^2^ |
| IQB675 | Δ*msmX*::*cat* Δ*amyE*::pSpank(hy)-*msmX*-(Lys43Ala) | pAM13 → IQB495 |
| ISN10 | Δ*msmX*::*cat* Δ*amyE::*Pspank(hy)*-msmX*-LEHis_6_-*spc* | pPS7 → IQB495 |
| ISN11 | Δ*msmX*::*cat* Δ*amyE::*Pspank(hy)*-ABC_Bt*-LEHis_6_-*spc* | pPS8 → IQB495 |
| ISN12 | Δ*msmX*::*cat* Δ*amyE::*Pspank(hy)*-msmK*-LEHis_6_-*spc* | pPS9 → IQB495 |
| ISN13 | Δ*msmX*::*cat* Δ*amyE::*Pspank(hy)*-ABC_Sa*-LEHis_6_-*spc* | pPS10 → IQB495 |
| ISN14 | Δ*msmX*::*cat* Δ*amyE::*Pspank(hy)*-malK*-LEHis_6_-*spc* | pPS11 → IQB495 |
| ISN15 | Δ*msmX*::*cat* Δ*amyE::*Pspank(hy)*-frlP*-LEHis_6_-*spc* | pPS12 → IQB495 |
| ISN17 | Δ*msmX*::*cat* Δ*amyE::*Pspank(hy)*-ycjV*-LEHis_6_-*spc* | pPS14 → IQB495 |
| ISN25 | Δ*msmX*::*cat* Δ*amyE::*Pspank(hy)*-ABC_Cd*-LEHis_6_-*spc* | pSN88 → IQB495 |
| ISN58 | Δ*msmX*::cat Δ*amyE*::Pspank(hy)-*ABC_Syn*- LEHis_6_-*spc* | pIG3 → IQB495 |

**Table 7 – Data collection and refinement statistics of MsmX K43A mutant.**

|  | MsmX K43A |
| --- | --- |
| **Data collection** |  |
| Wavelength (Å) | 0.9919 |
| Space group | I222 |
| **Cell parameters** |  |
| *a*, *b*, *c* (Å) | 86.76, 94.25, 132.26 |
| Resolution range (Å) | 45.93 – 1.67 (1.80 – 1.67) |
| *R*_pim_ (%) | 1.8 (52.3) |
| No. of observed reflections | 271,566 (13,552) |
| No. of unique reflections | 41,647 (2,082) |
| **Ellipsoidal resolution limits (Å)** |  |
| a^*^ | 1.63 |
| b^*^ | 2.42 |
| c^*^ | 1.71 |
| <I/σ(*I*)> | 18.0 (1.5) |
| Spherical completeness (%) | 65.9 (16.9) |
| Ellipsoidal completeness (%) | 95.0 (69.6) |
| Multiplicity | 6.5 (6.5) |
| **REFINEMENT** |  |
| Resolution (Å) | 19.97 - 1.67 |
| No. reflections | 38,380 |
| *R_work_/R_free_* | 0.20/0.25 |
| **No. atoms** |  |
| Protein | 2,962 |
| Ligand/ion | 15 |
| Water | 257 |
| <B-factor> (Å^2^) | 42.44 |
| **R.m.s deviations** |  |
| Bond lengths (Å) | 0.020 |
| Bond angles (^o^) | 1.956 |
| **Protein geometry** |  |
| Rotamers outliers (%) | 0.62 |
| Ramachandran outliers (%) | 0.00 |
| Ramachandran favored (%) | 97.53 |
| Cβ deviations > 0.25 Å (%) | 0.29 |
| Bad bonds (%) | 0.07 |
| Bad angles (%) | 0.12 |

Statistics for the highest-resolution shell are shown in parentheses. The dataset was anisotropy-corrected and merging statistics were calculated from ellipsoidally truncated data. Asterisks indicate reciprocal cell directions.

**SUPPLEMENTARY REFERENCES**

1. Lanzetta, P. A., Alvarez, L. J., Reinach, P. S. & Candia, O. A. An improved assay for nanomole amounts of inorganic phosphate. *Anal. Biochem*. **100**, 95–97 (1979).

2. Ferreira, M. J., Mendes, A. L. & Sá-Nogueira, I. The MsmX ATPase plays a crucial role in pectin mobilization by *Bacillus subtilis*. *PLoS One* **12**, 1–22 (2017).

3. Studier, F. W., Rosenberg, a H., Dunn, J. J. & Dubendorff, J. W. Use of T7 RNA polymerase to direct expression of cloned genes. *Method Enzymol* **185**, 60–89 (1990).

4. Sá-Nogueira, I., Nogueira, T. V., Soares, S. & Lencastre, H. de. The *Bacillus subtilis* L-arabinose (ara) operon: nucleotide sequence, genetic organization and expression. *Microbiology* **143**, 957–969 (1997).

5. Ferreira, M. J. & Sá-Nogueira, I. A multitask ATPase serving different ABC-type sugar importers in *Bacillus subtilis*. *J. Bacteriol.* **192**, 5312–8 (2010).

6. Krissinel, E. & Henrick, K. Secondary-structure matching (SSM), a new tool for fast protein structure alignment in three dimensions. *Acta Crystallogr.* **D60**, 2256–2268 (2004).

7. Chen, J., Lu, G., Lin, J., Davidson, A. L. & Quiocho, F. A. A tweezers-like motion of the ATP-binding cassette dimer in an ABC transport cycle. *Mol. Cell* **12**, 651–661 (2003).

8. Lu, G., Westbrooks, J. M., Davidson, A. L. & Chen, J. ATP Hydrolysis Is Required to Reset the ATP-Binding Cassette Dimer into the Resting-State Conformation. *Proc. Natl Acad. Sci. USA* **102**, 17969–1797 (2005).

9. Khare, D., Oldham, M. L., Orelle, C., Davidson, A. L. & Chen, J. Alternating Access in Maltose Transporter Mediated by Rigid-Body Rotations. *Mol. Cell* **33**, 528–536 (2009).

10. Oldham, M. L. & Chen, J. Crystal structure of the maltose transporter in a pretranslocation intermediate state. *Science.* **332**, 1202–1205 (2011).

11. Oldham, M. L., Khare, D., Quiocho, F. A., Davidson, A. L. & Chen, J. Crystal structure of a catalytic intermediate of the maltose transporter. *Nature* **450**, 515–521 (2007).

12. Ose, T., Fujie, T., Yao, M., Watanabe, N. & Tanaka, I. Crystal structure of the ATP-binding cassette of multisugar transporter from *Pyrococcus horikoshii* OT3. *Proteins Struct. Funct. Genet.* **57**, 635–638 (2004).

**Fig. 1: Genomic context of putative uncharacterized NBDs used in this study.**

The genomic context of each NBD was retrieved from NCBI Gene Database using the following gene IDs: 937070 (*frlP*), 14558340 (*ABC_Bt*), 12323215 (*ugpC*, *ABC_Sa*), 4914566 (*ABC_Cd*), 945890 (*ycjV*) and 14618496 (*ABC_Syn*). The respective coding proteins are available at NCBI with the following RefSeq: NP_391135.1, WP_000818931.1, WP_000818906.1, WP_011861561.1, WP_000057985.1 and WP_010874222.1.
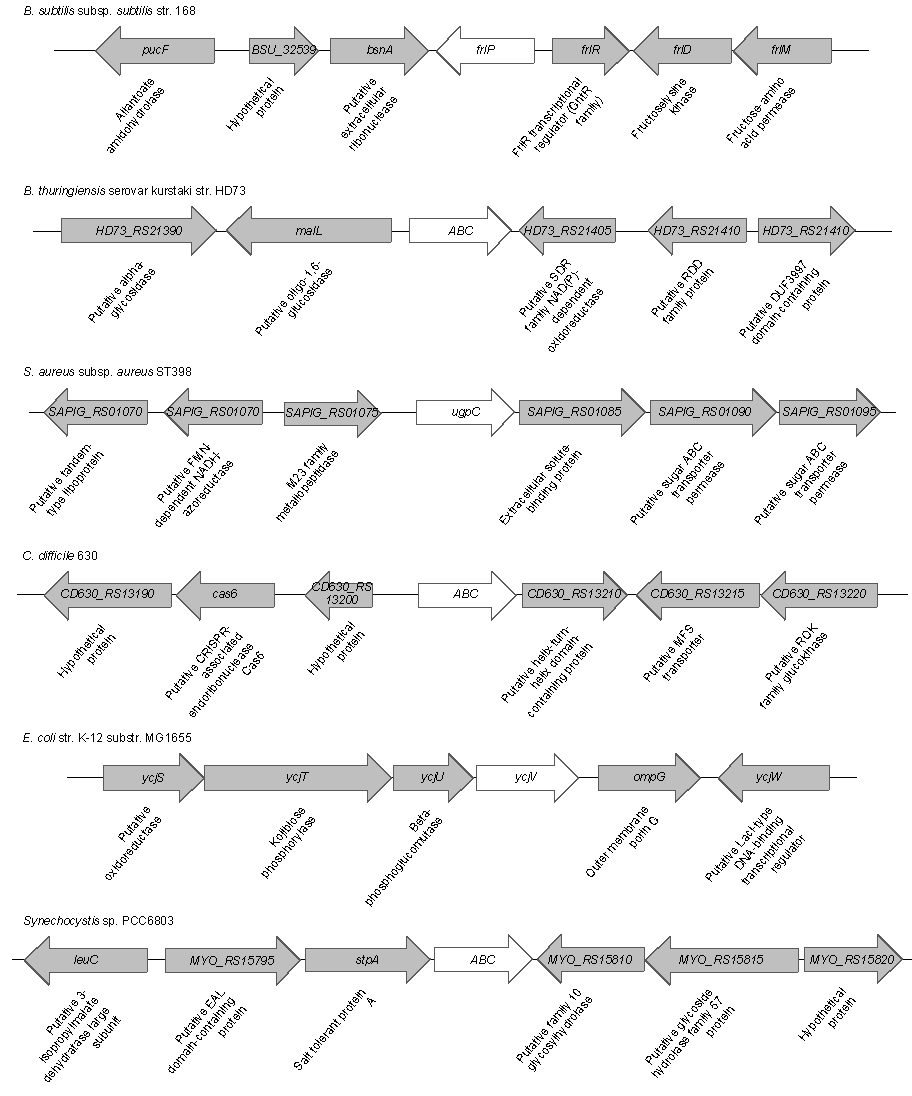


**Fig. 2: ATPase activity of MsmX-LEHis_6_, MalK-LEHis_6_, MsmX D77A/K154A-LEHis_6_ and YcjV-LEHis_6_**
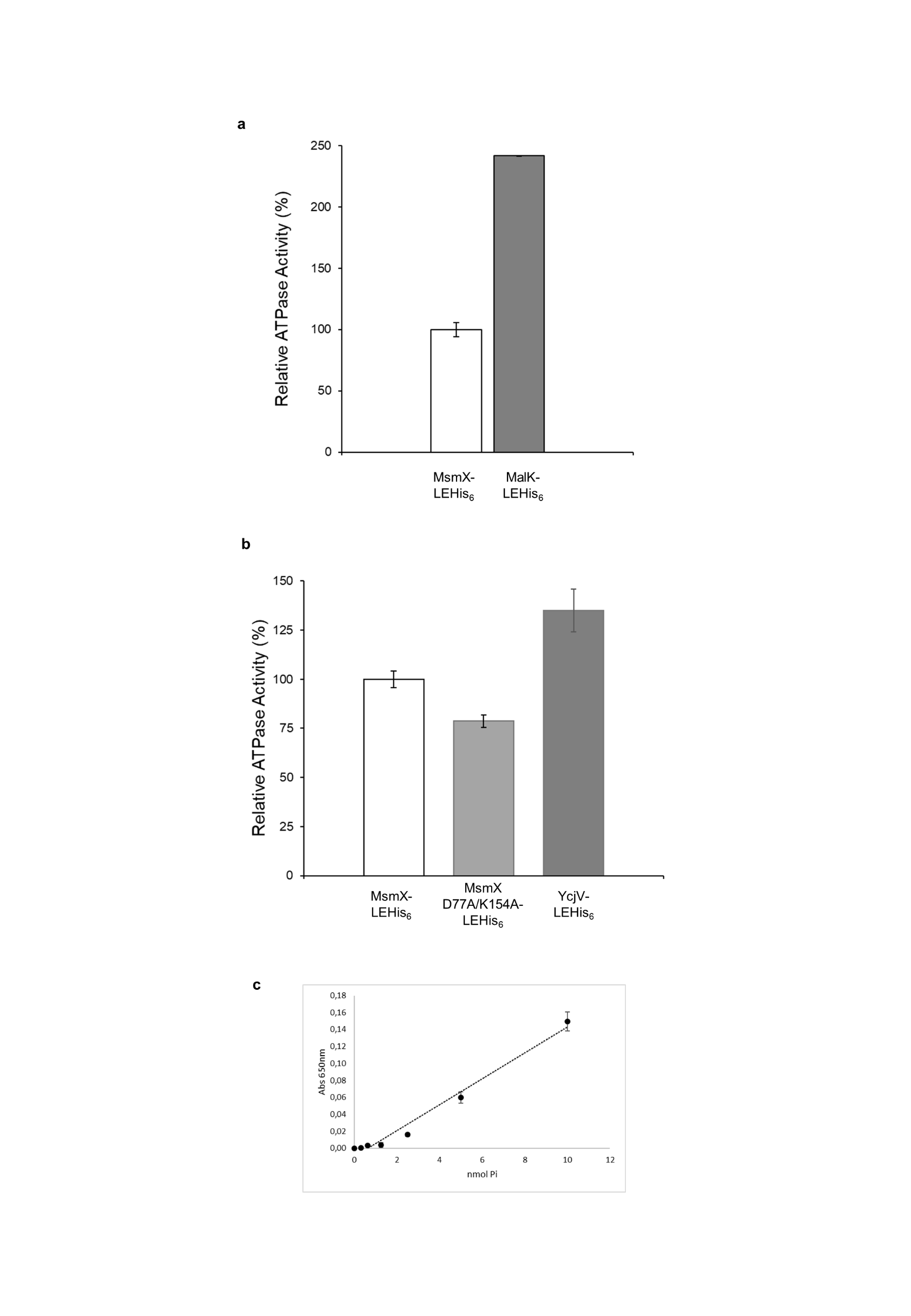


Endpoint ATPase assays of 4 h were performed to compare the enzymatic activity of MsmX-LEHis_6_, MalK-LEHis_6_, MsmX D77A/K154A-LEHis_6_ and YcjV-LEHis_6_. ATPase hydrolysis was calculated as µmol inorganic phosphate generated per hour per milligram of protein. ATPase activity of MalK-LEHis_6_ is presented relatively to MsmX-LEHis_6_, measured in triplicate in two independent assays. Both MsmX-LEHis_6_, MalK-LEHis_6_ were obtained from the soluble protein fraction as described previously in the section Expression and purification of recombinant ATPases (**a**). MsmX D77AK/154A-LEHis_6_ and YcjV-LEHis_6_, as well as MsmX-LEHis_6_, for comparative purposes, were obtained from the insoluble fraction of protein by denaturation with 3 M Guanidine HCl followed by renaturation inside the column, as described in the section Expression and purification of recombinant ATPases (**b**). Calibration of free inorganic phosphate is shown. The mean results with standard deviations are displayed for all measurements (**c**).

**Fig. 3: ATPase activity of MsmX is impaired by K43A mutation but not abolished**

**
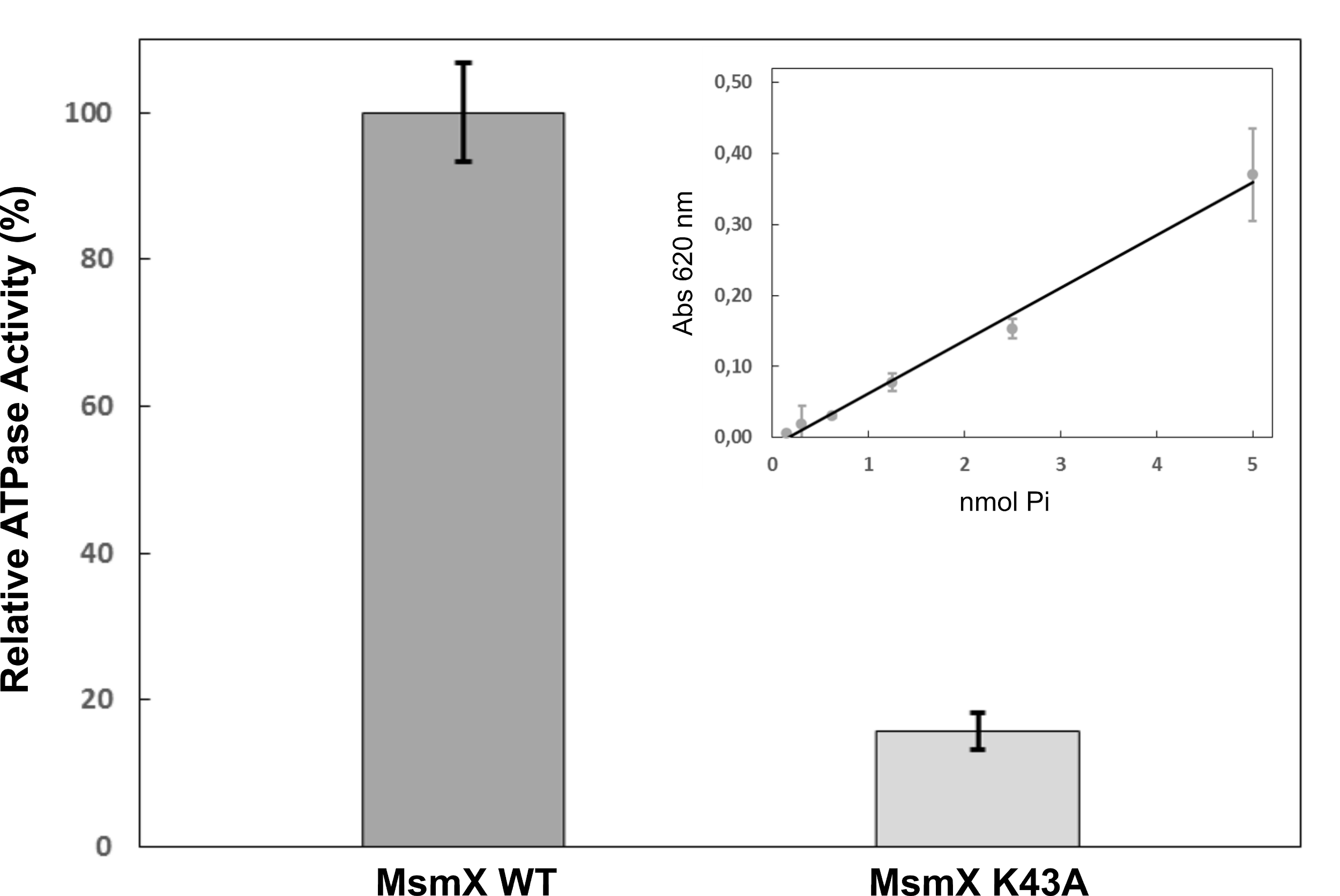
**

Endpoint ATPase assays of 4 hours were performed to compare the enzymatic activity of MsmX WT and MsmX K43A. ATPase hydrolysis was calculated as µmol inorganic phosphate generated per hour per milligram of protein, and the ATPase activity is presented relatively to MsmX WT. The calibration of free inorganic phosphate is shown in the inset figure. The mean results with standards errors are displayed for all measurements.

**Fig. 4: Accumulation of MsmX-His_6_ in the presence of arabinose and arabinotriose.**


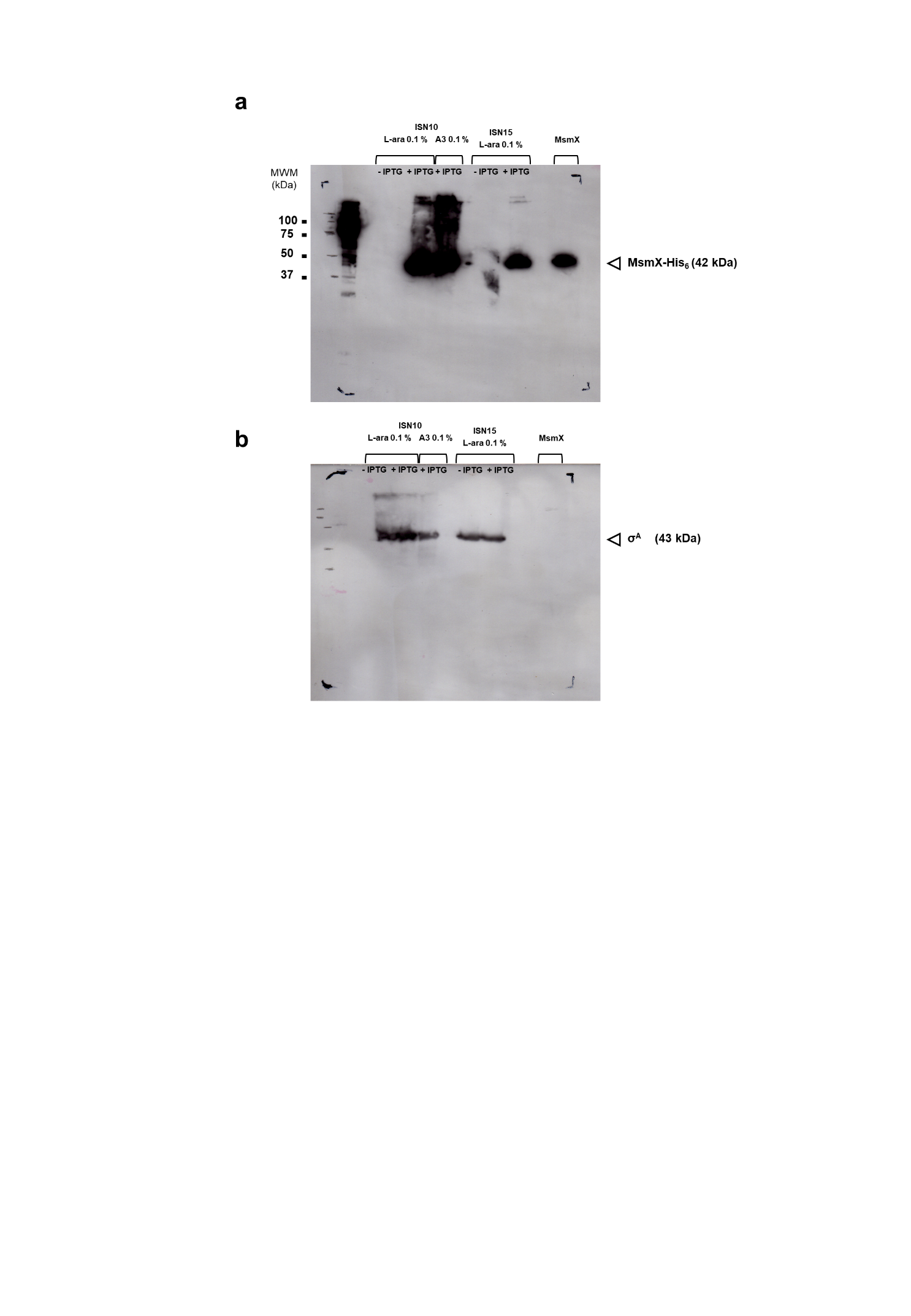


Western blot analysis of MsmX-His_6_ accumulation in total cell extracts of *B. subtilis* strain ISN10 in the presence of arabinose (L-ara) or arabinotriose (A3) as sole carbon and energy source. Accumulation of FrlP-His_6_ (strain ISN15) after IPTG induction in the presence of arabinose was also analyzed (a). Normalization of total protein was assessed using the anti-σ^A^ antibody (b). Purified MsmX-His_6_ (0.25 μg) was loaded on the last lane. Precision Plus Protein All Blue Prestained Protein Standard (Bio-Rad) was used. These results show that MsmX accumulation upon induction with 1 mM IPTG is comparable both in the presence of L-ara or A3.

**Fig. 5: Uncropped Western blots from Figure 2c.**
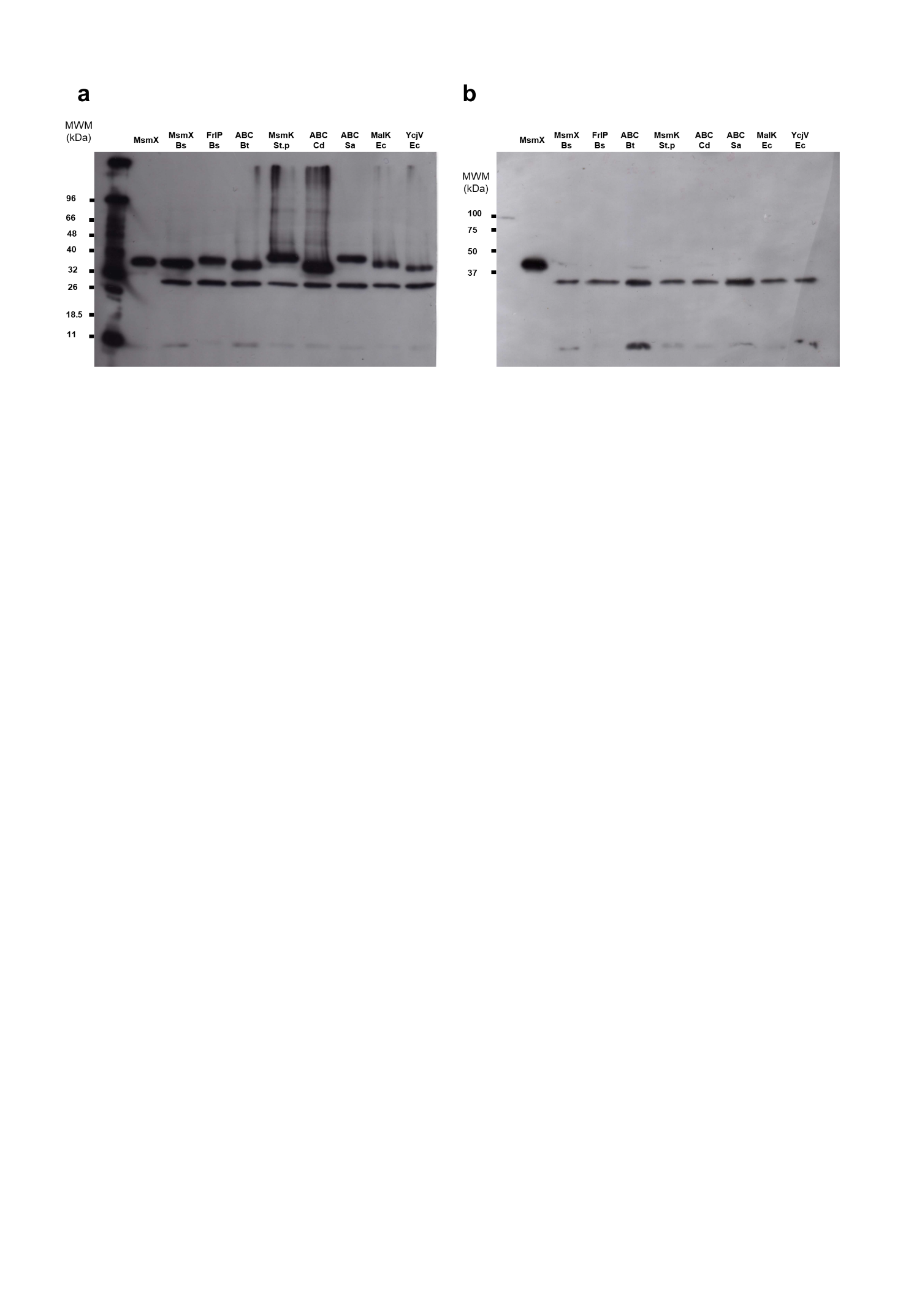


Western blot analysis of NBD accumulation in total cell extracts. This corresponds to the full blot presented in Figure 2c, which was cut for clarity purposes. NBD accumulation in total cell extracts of each strain grown in the presence (**a**) or absence (**b**) of IPTG. Low Molecular Weight, Protein Marker II (NZYTech) (**a**) or Precision Plus Protein All Blue Prestained Protein Standard (Bio-Rad) (**b**) were used.

**Fig. 6: Uncropped Western blots from Figure 3c**
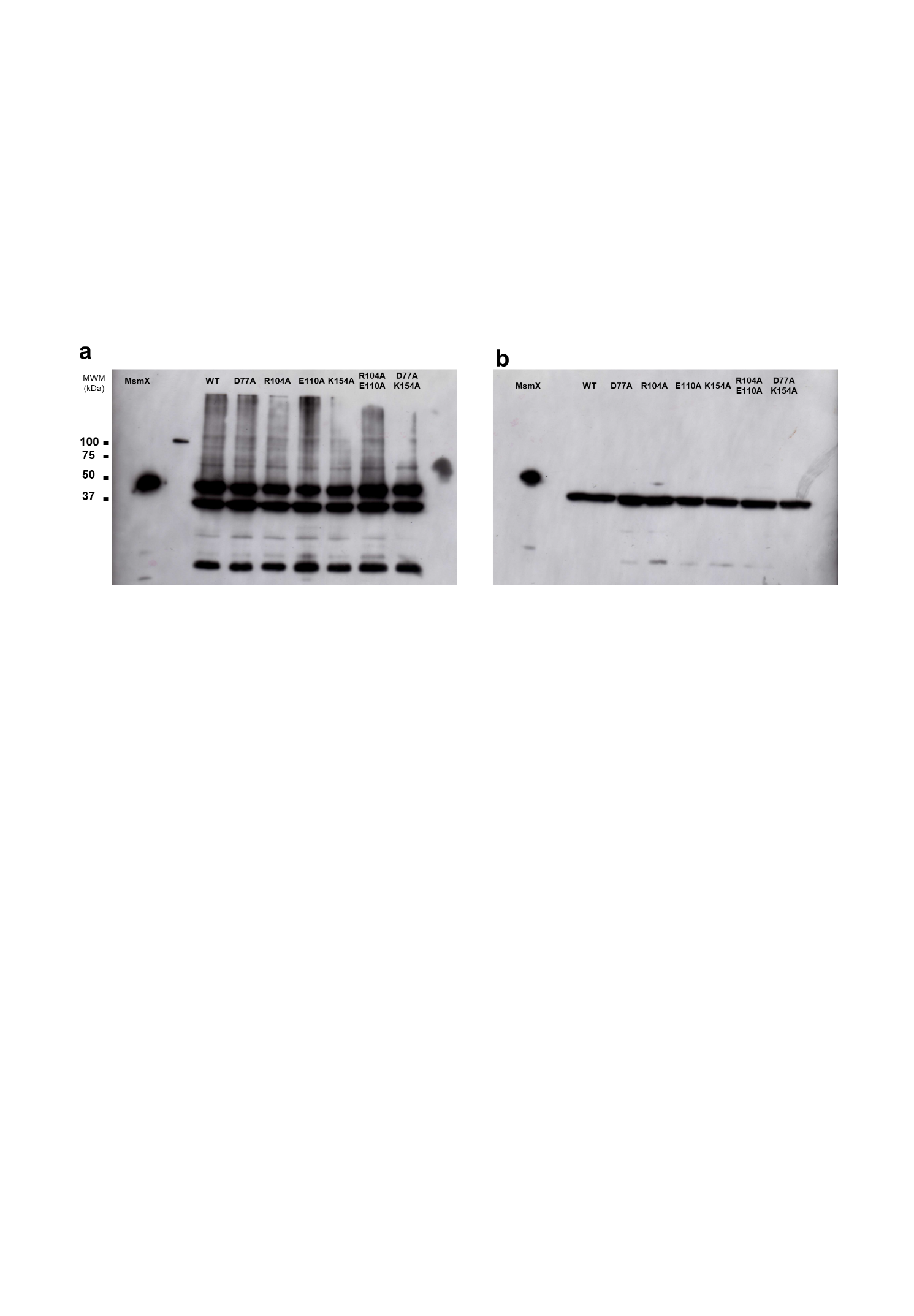


Western blot analysis of MsmX and MsmX mutant variants accumulation in total cell extracts. This corresponds to the full blot presented in Figure 3c, which was cut for clarity purposes. MsmX mutant variants accumulation in total cell extracts of each strain grown in the presence (**a**) or absence (**b**) of IPTG. Precision Plus Protein All Blue Prestained Protein Standard (Bio-Rad) was used.

**Fig. 7: Uncropped Western blots from Figure 4b**

**
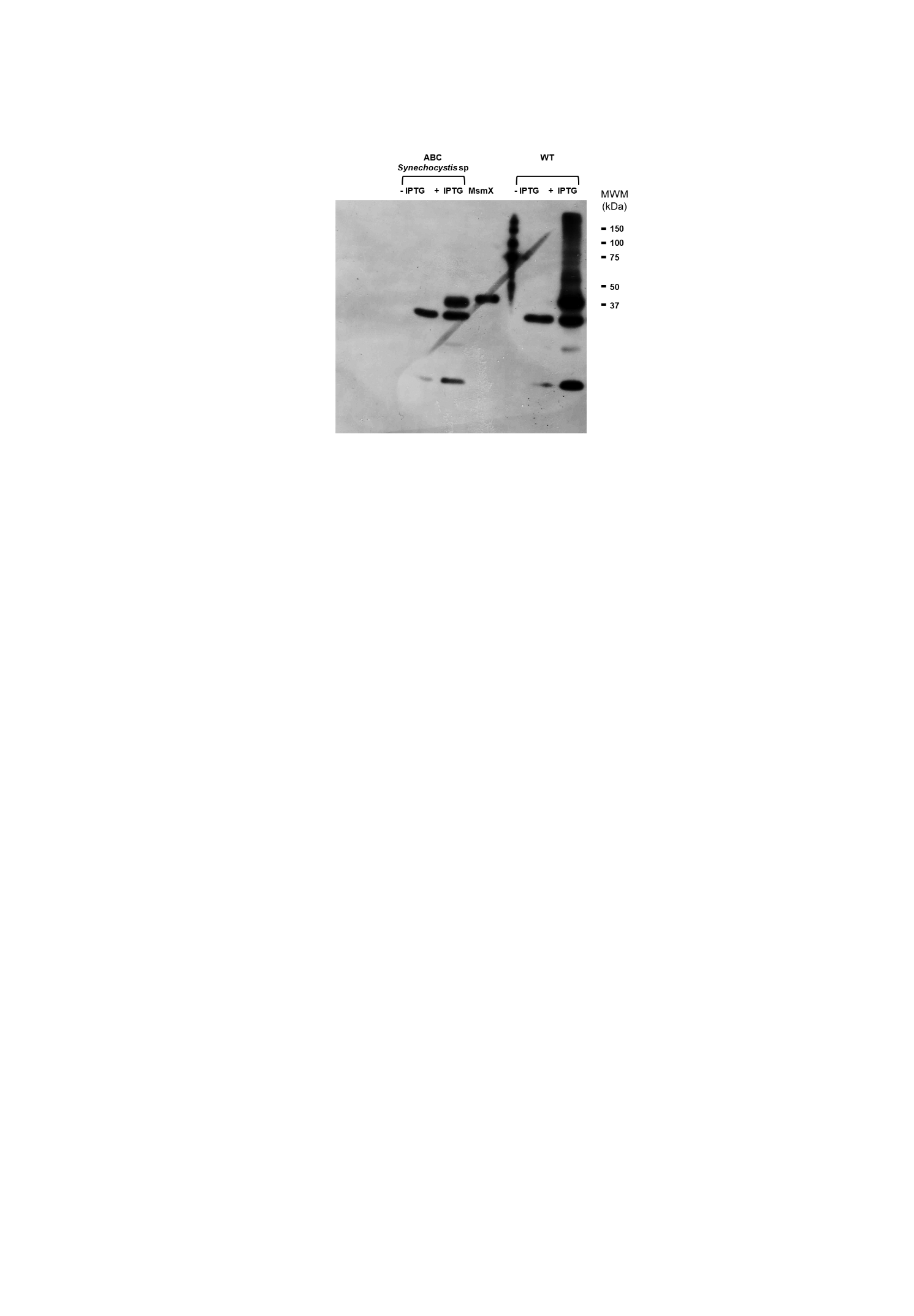
**

Western blot analysis of Western blot analysis of NBD accumulation in total cell extracts of *Synechosystis* sp grown in the absence (-) or presence (+) of IPTG. This corresponds to the blot presented in Figure 4b, which was cut for clarity purposes Precision Plus Protein All Blue Prestained Protein Standard (Bio-Rad) was used.
